# A Bayesian Hierarchical Model for Estimation of Abundance and Spatial Density of *Aedes aegypti*

**DOI:** 10.1371/journal.pone.0123794

**Published:** 2015-04-23

**Authors:** Daniel A. M. Villela, Claudia T. Codeço, Felipe Figueiredo, Gabriela A. Garcia, Rafael Maciel-de-Freitas, Claudio J. Struchiner

**Affiliations:** 1 Fundação Oswaldo Cruz, Programa de Computação Científica—Rio de Janeiro, Brazil; 2 Fundação Oswaldo Cruz, Departamento de Entomologia, Laboratório de Transmissores de Hematozoários—Rio de Janeiro, Brazil; Swedish University of Agricultural Sciences, SWEDEN

## Abstract

Strategies to minimize dengue transmission commonly rely on vector control, which aims to maintain *Ae. aegypti* density below a theoretical threshold. Mosquito abundance is traditionally estimated from mark-release-recapture (MRR) experiments, which lack proper analysis regarding accurate vector spatial distribution and population density. Recently proposed strategies to control vector-borne diseases involve replacing the susceptible wild population by genetically modified individuals’ refractory to the infection by the pathogen. Accurate measurements of mosquito abundance in time and space are required to optimize the success of such interventions. In this paper, we present a hierarchical probabilistic model for the estimation of population abundance and spatial distribution from typical mosquito MRR experiments, with direct application to the planning of these new control strategies. We perform a Bayesian analysis using the model and data from two MRR experiments performed in a neighborhood of Rio de Janeiro, Brazil, during both low- and high-dengue transmission seasons. The hierarchical model indicates that mosquito spatial distribution is clustered during the winter (0.99 mosquitoes/premise 95% CI: 0.80–1.23) and more homogeneous during the high abundance period (5.2 mosquitoes/premise 95% CI: 4.3–5.9). The hierarchical model also performed better than the commonly used Fisher-Ford’s method, when using simulated data. The proposed model provides a formal treatment of the sources of uncertainty associated with the estimation of mosquito abundance imposed by the sampling design. Our approach is useful in strategies such as population suppression or the displacement of wild vector populations by refractory *Wolbachia*-infected mosquitoes, since the invasion dynamics have been shown to follow threshold conditions dictated by mosquito abundance. The presence of spatially distributed abundance hotspots is also formally addressed under this modeling framework and its knowledge deemed crucial to predict the fate of transmission control strategies based on the replacement of vector populations.

## Introduction

Dengue fever is the most prevalent arbovirus infection in the world with 2.5 billion people living in areas under the risk of transmission [1]. The temporal and spatial pattern of dengue distribution is influenced by mosquito, human, viral and environmental factors. The abundance, survival and dispersal of its vector, the *Aedes aegypti* mosquito, are important factors to describe the ecology involving the mosquito. In particular, spatial distributions of mosquito populations at fine scales [2, 3] can help understand the impact on dengue transmission. Estimation of parameters that describe these components is still a complex challenge for field entomologists and modelers [4–7].

Knowledge about these key components is crucial [8] in guiding vector control strategies based on population suppression in areas at transmission risk. Several dengue endemic countries, including Brazil, plan their vector control strategies based on the assessment of infestation indices through larval surveys, most commonly House and Breteau Indices (HI and BI, respectively) [9, 10]. These traditional infestation indices show low correlation with adult mosquito abundance, as HI and BI do not consider container productivity and larval mortality [10, 11]. Alternatively, trapping of adults with a variety of devices has been proposed as a more efficient approach to monitor *Aedes aegypti* populations and many initiatives are already in place worldwide. Traps allow the development of more standardized protocols, provide indices faster and require less effort than the traditional searching approach. One drawback of a trap based infestation index, however, is that it is a relative measure of population density, with unit mosquito/trap. For comparative purposes, this may suffice. But there are situations when absolute measures of population abundance (unit: mosquitoes/area or mosquitoes/person) are of interest. For example, vector thresholds for transmission are defined in terms of mosquito/person [8]; population thresholds for *Aedes aegypti* + *Wolbachia* invasion is defined in mosquito/area [12, 13].

Traditionally, the estimation of animal population size is performed via experiments of mark, release and recapture (MRR). In MRR experiments, subjects are typically captured from the environment, marked either uniquely or as a cohort using a piece of identification (tags, colors etc.), released back into the environment and later recaptured, possibly multiple times. For this kind of experiment, mosquitoes are challenging subjects due to their small size and short life span, making recapturing difficult, which led to important modifications of the standard protocols, mainly, that marked mosquitoes are released and subsequently recaptured only once [7]. Models for estimating adult population size from MRR datasets, either deterministic or stochastic ones, include Lincoln, Jolly-Seber, and Fisher and Ford [6, 7, 14–16]. The Lincoln Index, due to its simplicity, is often the method of choice. However this simplicity comes at a price, since the strong assumptions required for the proper utilization of this index are difficult to meet in most field conditions, for example, that the population is closed and that there is no heterogeneity in capture rates. The Fisher—Ford method relaxes some of these assumptions allowing the loss of individuals by mortality. These models are not probabilistic and do not treat sampling uncertainty properly. The Jolly-Seber family of models is very popular due to their probabilistic framework, however they are tailored for data with multiple recaptures and fitting with typical mosquito data does not show convergence (results not shown). In this work we investigate the potential advantages to analyze MRR mosquito trials of a class of stochastic models containing an ecological component introduced by Royle *et al*. [17]. Stochastic models involve more complex mathematical calculations, but generate estimates of population size along with measures of uncertainty [7]. The model presented here was developed upon the basic structure of the model of Royle *et al*. [17] to accommodate particular aspects of the mosquito ecology and its observation. We perform Bayesian analysis using this model to estimate abundance and spatial distribution of the population of female *Aedes aegypti* in a dengue endemic area in the city of Rio de Janeiro, Brazil. The proposed model is also tested with artificial data from a simulator of typical mosquitoes’ MRR experiments.

In the next section we describe the MRR experiments carried out in the study area in Rio de Janeiro, Brazil. The description of the experiments is helpful here as it illustrates the particular aspects of a typical MRR dataset for mosquitoes. We then describe the structure of the hierarchical model, its layered components and the simulation environment. We present estimates of abundance, spatial density and survival probability of *Aedes aegypti* in the study area comparing the estimates from the proposed hierarchical model with those from the Fisher—Ford method. Subsequently, we show some statistical properties of the proposed model obtained from its application to simulated data.

## Methods

### Mark—Release—Recapture Experiments

**Study area.** We conducted mark-release-recapture studies in a 7.2 ha neighborhood called Z—10, located in the Governador Island, city of Rio de Janeiro, Brazil. Z—10 (22°52′30″*S*; 43°14′53″*W*) is an isolated suburban community, surrounded by shores and mangroves, which discourage mosquito migration through its limits. This residential area has paved streets, regular sidewalks and low-moderate vegetation coverage, with around 2,350 people living in 787 houses. Most houses have 2–3 bedrooms, regular water supply and garbage collection, lacking peridomestic areas with pools or yards.

**Climate and MRR periodicity.** The climate in Rio de Janeiro is characterized by a moderately dry winter (May-August) and a wet summer season (November—March), with mean temperatures of 25.1°C and 28.8°C and mean total rainfall of 46.4 mm and 132 mm, respectively. MRR experiments were conducted during nine consecutive days in September 2012 and during another period of nine days in March 2013. In Sep/2012, temperatures ranged between 22.3°C and 30.6°C (average of 28.6°C) with no rainfall. In Mar/2013, temperatures ranged from 23.1°C to 32.5°C, with an average of 29.7°C and rainfall of 0.4 mm. Air temperature and precipitation data were obtained from a meteorological station located at approximately 5 km away from the study area.

**Mosquitoes.** *Aedes aegypti* adults used in MRR experiments were derived from the F1 generation of eggs collected at Z—10 using 60 ovitraps filled with hay infusion. Larvae were fed with fish food (Tetramin, Tetra Sales, Blacksburg, VA) and reared according to Consoli and Lourenço-de-Oliveira [18]. After emergence, females were kept together at 25±3°C and 65±5% relative humidity (RH) and fed with sucrose solution until the time to release.

**Marking and releasing.** Before releasing in Sept/2012, *Ae. aegypti* adults were split into four cohorts, each one composed of 500 females marked with different colors of fluorescent dust (Day-Glo Color Corp., Cleveland, OH) and placed in small cylindrical cups (12 cm x 10 cm). Each cohort was released from a different outdoor location in Z—10. In Mar/2013, only one cohort composed of 2000 females was released in a central outdoor location of Z—10. In both occasions, mated, unfed females (4—day old) were released in the morning hours (between 8:00 AM and 9:00 AM), at approximately 1 hour after dust marking. Wind direction and speed was 139.7° and 4.7 m/s in the moment of release in Sep/2012. Meanwhile, in Mar/2013, wind direction and speed was 25.1° and 2.6 m/s.

**Capturing.** Dust-marked females were captured using 66 uniformly distributed BG-Sentinels traps (BioGents, Regensburg, Germany), designed to attract mosquitoes seeking a host to blood feed [19, 20]. Captures started at the following day after mosquitoes’ release and was performed daily by inspection of the 66 BGs-Traps. Daily capturing stopped when no dust-marked females were collected for 3 consecutive days. Captured mosquitoes were examined under UV light to check for the presence of fluorescent dust.

#### Ethics Statement

Fiocruz conducts regular research activities in the city of Rio de Janeiro, in partnership and also with permission of the Rio de Janeiro Department of Health (*Secretaria de Saúde da cidade do Rio de Janeiro*) that assist in the control of transmission of infectious diseases. Mark-release-recapture experiments were approved by Fiocruz Ethical Committee (CEP 253/04) and carried out as part of this research effort, not requiring a specific permission. Since mosquitoes are released in public open spaces such as squares, there was no need to obtain individual consent for releases. In order to recapture marked mosquitoes, inside people’s dwellings, we have to obtain consent from residents to install BG-Traps and also to carry daily collections after the release of marked individuals. The use of mosquito traps for sampling *Aedes aegypti* belongs to the routine of vector surveillance in Rio de Janeiro. Due to this surveillance routine, many residents in the study area are used to have mosquito traps installed in their houses and, indeed, there were just few refusals by residents to have traps installed. Written consent was not required under the ethical committee requirements. As the effort to collect mosquitoes goes along with city surveillance, these tasks are typically done with verbal consent only and our collection effort also had verbal consent, as approved by the ethics committee, and individual consents were registered by the entomology field team in regular work files.

The release of mosquitoes does not involve directly endangered or protected species and, from our experience, it does not have any significant impact on endangered or protected species. One of our main concerns during MRR experiments is related to dengue transmission by the mosquitoes released in the field. In order to address this issue, we follow two guidelines. The first one is to not release a number of mosquitoes greater than the number of mosquitoes removed by trapping in pilot experiments and monitoring activities. Second, the procedure is to interrupt or to suspend, depending on circumstances, mosquito releases if dengue cases are notified in that neighborhood. Since both trap inspection and mosquito collection are done with the assistance of the health department in the city government, i.e., the same agency that is responsible for dengue notification, dengue cases are rapidly identified.

### Hierarchical Model

The proposed model has three components. The first component is a probabilistic model describing the spatial distribution of mosquitoes in the study area, while the second component is also a probabilistic model describing the daily survival of marked and native mosquitoes. Finally, the observation model describes the sampling process.

**Ecological process—center of activities.** There is some evidence that mosquitoes tend to remain close to their birth location if conditions for blood feeding and ovipositing are adequate. Specialists typically agree that *Ae. aegypti* females rarely visit more than 2–3 houses during their lifetime leading to a strong spatial correlation between the distribution of immature and adults [21]. Harrington *et al*. [4] reviewed several MRR experiments carried out in different locations, different release sites (both indoors and outdoors) and experimental protocols. Overall, these studies agree on the limited dispersal behavior of *Aedes aegypti*. When release is done indoors, most of the collections occur in the same house [22, 23]. This tendency to remain in the same location (at least during the length of the experiment) can be formalized by the concept of center of activity. An individual center of activity is a central point (centroid) of the space occupied by the individual during a time interval [24]. This concept is borrowed from the works of Royle *et al*. [17, 25], in which they model the movement of tigers [17] and birds [25].

Here, there are two important claims. First, we assume that for the duration of the MRR experiments spatial density of mosquitoes can be described by the density of activity centers, as mosquitoes tend to stay within a constrained space. This is reasonable for both marked and unmarked individuals given the empirical observations as described above. Second, the marked mosquitoes are released and go through a dispersal phase. Here, we have evidence that the dispersal is fast and subsequent captures of individuals will find them already in the whereabouts of their center of activities. In the MRR experiment studied here, marked cohorts were released outdoors, in locations that stimulate dispersal towards more suitable habitats. Under stress mosquitoes can fly more than 600 m for three days to find suitable conditions [26]. Evidence for the ability to reach the whole area comes from the observation that in the first day of capturing, marked mosquitoes were already found in the most distant traps. Therefore, we distinguish between the short distance movements close to centers of activity and the long range movements realized when dispersal happens.

In the model the center of activity is given by a pair **s** = (*s*_*x*_, *s*_*y*_) of coordinates that describe the center of an individual area. The prior distribution for these pairs of coordinates is generally a uniform distribution. After a procedure of inference, given the observations in the MRR experiments, the distribution of center of activities is effectively evaluated as a spatial density distribution within the study area.

**Ecological process—survival.** *Aedes aegypti* life span ranges from 5 to 30 days [4, 27–31], making survival an important process to be considered in an experiment that lasts for 9 days. In the capture data (shown in the Results section), there is clearly a loss of marked individuals as days go by. This loss can be attributed to mortality by natural causes. In the hierarchical model, there is a component that describes daily survival probability *ϕ*, assumed equal across all marked individuals. For the unmarked individuals, however, under an assumption that abundance stays at a stable level for the relatively short length of an MRR study, we consider that recruitment cancels out mortality, *i.e*., in terms of the model, *ϕ* = 1 for unmarked individuals.

**Observation process.** In the model each trap has associated with it a probability of capturing mosquitoes as a function of the distance between the trap location, recorded in the experiment, and any point in the study area. Hence, each trap has a probability of capturing the mosquito as function of the distance between the trap location and its center of activity. Following the approach in [17], the probability to be attracted to a trap is described by a function that has parameters to be estimated as a *Generalized Linear Model* (GLM). We choose the complementary log—log function as a link function, thus we have for the probability *π*_*i*,*j*_ of individual *i* be attracted to trap *j*, *cloglog*(*π*_*i*,*j*_)∣**s_i_** ∼ *β*_0_ + *β*_1_ *d*_*i*,*j*_, where *d*_*i*,*j*_ is the distance from individual *i*’s center of activity and trap *j*’s location, and *β*_0_ and *β*_1_ are parameters to be estimated.

The observation model takes into account that each individual can only be trapped once. This limitation impacts the model formulation in two main aspects. First, at each observation time point (intervals given in days and total time *T*) the probability of an individual being captured at a particular trap is a product of two factors: the probability *π*_*i*,*j*_ of individual *i* to be captured at trap *j*, given by a function of the distance from its center of activity to the trap, and the probability to be captured at trap *j*, given by a categorical random variable with parameter vector given by the ratio $\frac{\pi_{i}}{\sum_{j=1}^{J}\pi_{i,j}}$. Once a marked individual is captured, it is removed from the study. Therefore, it cannot be observed again. In the model, removal is introduced in the survival component as a variable that indicates presence in the study. For each individual, this presence depends on its survival and also on the individual not being captured. Captures of mosquitoes at traps are described by the observation variables *y*_*i*,*j*,*t*_, indicating whether a mosquito *i* is observed (*y*_*i*,*j*,*t*_ = 1) or not (*y*_*i*,*j*,*t*_ = 0) at trap *j*, *j* ≤ *J*, at time *t*, *t* ≤ *T*.

We want to estimate the number *N*_2_ of unmarked mosquitoes in the study area using the hierarchical model in which each individual is indexed, whereas the number *N*_1_ of marked mosquitoes released in the study is known. Without any loss of generality, the first *N*_1_ individuals are the marked ones. We use the data augmentation technique [32] as used by Royle and Dorazio in [33] to estimate the number of unmarked mosquitoes *N*_2_. The technique involves taking a number *M* > *N*_1_ + *N*_2_ by adding non-existing individuals, since we do not know *N*_2_. The non-existing individuals will not be present in the study and will not be captured (zero values in observation). This requires another layer in the model to treat the zero—inflated component, that distinguishes zeroes from zero—inflation to zeroes due to non-observations. This component uses a set of variables *w*_*i*_, 1 ≤ *i* ≤ *M*, *M* > *N*_1_ + *N*_2_, each of which describes whether a particular individual *i* is effectively in the study population (either marked or unmarked population). Finally, this technique permits us to have abundance as an indirect measure obtained from an inference procedure.

The model is described in detail in S1 Text, including a table that describes the model and each of the components.

### Estimation and Bayesian Inference

Inference is performed via analysis of Monte—Carlo Markov Chain (MCMC) simulations. The abundance, *i.e*., the number *N*_2_ of unmarked individuals is estimated as a latent variable:

$$\begin{array}{c} N_{2}=\sum_{i=N_{1}+1}^{M}w_{i}. \end{array}$$ (1)

Since the estimation of variables is performed using multiple runs of MCMC simulations, results are generally given by statistical measures that include mean, median, standard deviation and credibility intervals.

The spatial density of mosquitoes is found using the posterior samples from MCMC simulation and constructing a grid over the study area. The count of individuals in each of the grid cells is found by the sum of center of activities inside each of them, similar to counts performed by Royle *et al*. in [17].

In order to find estimates, we use JAGS [34], a Bayesian analysis tool that implements MCMC through Gibbs sampling and uses a model specification very similar to another popular tool, WinBUGS. The JAGS model is included in S1 Text. The prior distribution for survival probability *ϕ*, defined in the [0, 1] interval, places higher weight on values closer to one, while also weakening extreme values in the [0, 1]—interval. This distribution is in accordance with empirical estimates of the survival rates of *Aedes aegypti* [35].

**Fisher—Ford method.** We also find estimates of abundance using Fisher—Ford method [36], which has been used in MRR analyses and is closely related to the Lincoln index (MRR on estimating abundance of *Aedes albopictus* by Cianci *et al*. [6] and *Anopheles gambiae* by Baber *et al*. [37]). The abundance estimator derives from the same argument of the Lincoln index, including an adjustment that takes into account the survival probability *ϕ* for marked individuals:

$$\begin{array}{c} {\hat{N}}_{2}=\frac{n_{tot}\phi^{t}N_{1}}{m_{tot}}-\phi^{t}N_{1}, \end{array}$$

where *n*_*tot*_ and *m*_*tot*_ are, respectively, the numbers of unmarked and marked individuals, captured at time *t*.

Bailey in [38] propose a bias correction in the case of small number of captures (typically below 20): ${\hat{N}}_{2}=\frac{\left(n_{tot}+1)\phi^{t}(N_{1}+1\right)}{m_{tot}+1}-\phi^{t}(N_{1}+1).$ We take a bootstrap approach in order to find a confidence interval for the Fisher—Ford index, using a number *R*_*s*_ of re—sampling.

**Simulation.** To assess the efficiency of the MRR experiment in various scenarios, an Individual-based model (IBM) was developed to simulate the dispersal and capture of marked mosquito cohorts, as a computational tool, written in the Perl programming language, that mimics the field experiments. Using this type of simulation environment it is possible to observe actual emergent phenomena from simple rules and it has been gaining attention in theoretical ecology since the last two decades [39–41].

The simulator is implemented by constructing classes that define objects for each agent, including agents for mosquitoes and traps, and it also defines a square area for the environment. For each mosquito, dispersal can be considered either random or towards a randomly chosen center of activity (sampled from a uniform distribution). Traps can be positioned at random locations, or at points comprising a centralized grid. In simulations we have the possibility of varying the area around which the trap attracts mosquitoes and also possibility to define the probability that describes a Bernoulli random variable to indicate mosquito capture at traps.

The output of the simulator is given by tables discriminating trap and mosquito data as well as a capture history file typical for MRR analysis programs, such as the program MARK [42]. Full descriptions for the objects, data structures and validation are provided in S2 Text. The code (open-source license—GNU PGL v3) is available from https://launchpad.net/mmrrsim.

## Results

### Field data from MRR experiments

Table 1 shows the number of daily captures observed in each of the mark—release—recapture experiments, conducted in Sept 2012 (ST1) and March 2013 (ST2). Data are shown separated by each of the cohorts (marking color) and the number of captures of unmarked mosquitoes is also shown. The counts of marked individuals generally decrease over time. For marked individuals the capture ratio is visibly higher in the days immediately subsequent to releasing in the field and has a decreasing trend along with time. The number of captures of unmarked individuals, however, tends to remain at a constant level. For the experiment in Sept. 2012, the ratio between total number of captures per number of released individuals varied from 6% (green cohort) to 13.4% (blue cohort), and including all cohorts the return capture ratio is 9.2%. For the experiment in March 2013, the capture ratio is 7.6%. Datasets are available as supporting information (S1 Dataset and S2 Dataset).

**Table 1:** Numbers on captures obtained from the two MRR experiments conducted in the Z—10 region in Rio de Janeiro, Brazil. ST1 refers to the study realized in Sept. 2012 and ST2 to the study realized in March 2013. For ST1, we present data on each of the four cohorts as indicated by the marked colors. For each of the studies, unmarked refers to the number of unmarked individuals captured.

Cohort	Num. Released	Day1	Day2	Day3	Day4	Day5	Day6	Day7	Day8	Total	Ratio
ST1—b	500	19	14	7	9	9	4	3	2	67	0.134
ST1—p	500	27	11	4	2	1	2	5	0	52	0.104
ST1—y	500	10	10	3	4	3	1	1	3	35	0.07
ST1—g	500	9	7	3	5	4	0	0	2	30	0.06
ST1—unmarked	—	15	22	12	17	36	14	25	21	162	—
ST2—b	2000	52	26	20	23	4	5	13	8	151	0.076
ST2—unmarked	—	119	92	95	64	93	114	110	99	786	—

In September 2012, we also measured size of the female wings from both populations (lab—reared and wild ones). Lab-reared *Ae. aegypti* females had 2.92 mm ± 0.085 mm (mean ± SD), meanwhile wild mosquitoes in this period had 2.74 mm ± 0.12 mm.

### Analysis of field data

To study the impact of increasing sample size on the abundance estimation, data from ST1 was pooled by using a separate cohort, defined by the marking color, or a combination of cohorts: cohort b (*N*_1_ = 500), cohorts b+p (*N*_1_ = 1000), cohorts b+p+y (*N*_1_ = 1500) and cohorts b+ p+ y+g (*N*_1_ = 2000).

**Fig 1:**
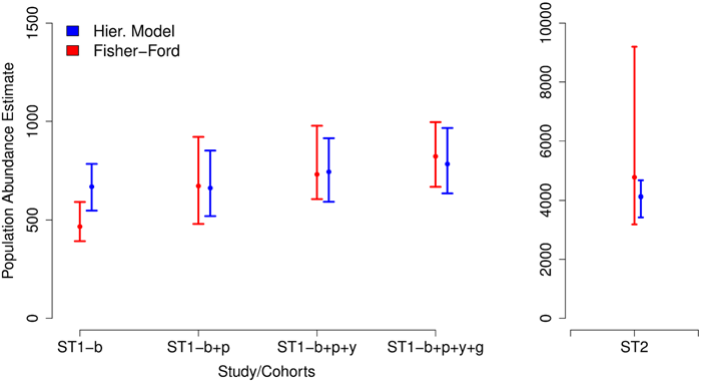
Results from estimation using data from field studies. Intervals colored in solid red indicate Fisher—Ford estimates. Intervals colored in solid blue indicate estimates from the hierarchical model. Labels ST1 and ST2 indicate studies conducted in September 2012 and March 2013, respectively. The September 2012 study had 4 cohorts given by 4 different colors: blue (b), pink (p), yellow (y) and green (g). Labels ST1 also include which cohorts were used in the analysis by grouping cohorts ({b}, {b+p}, {b+p+y}, { b+p+y+g}). Estimates of population abundance are shown along with both *y*—axes (different scales), the one on the left side for study ST1 and on the right side for study ST2.

Table 2 summarizes the pooled data and provides the abundance estimates found using the hierarchical model and the Fisher—Ford method. Fig 1 and Table 2 show the *Aedes aegypti* abundance estimates, according to both models.

**Table 2:** Estimates of abundance for the populations of female *Aedes aegypti* in Z—10, Rio de Janeiro, in Sept 2012 (ST1) and March 2013 (ST2), according to the hierarchical model and the Fisher-Ford model. Estimation realized using samples from 16000 iterations using data from both studies. The hierarchical model results are obtained using JAGS after running 10000 iterations per chain (first 2000 were discarded) in two Markov chain simulations. Fisher—Ford estimates are obtained using a bootstrap approach (re-sampling *R*_*s*_ = 1000). For the Fisher—Ford estimation the survival rate is assumed to be *ϕ* = 0.8.

	Hierarchical Model				Fisher—Ford	
Cohort	Mean	Std. dev.	Median	95% CI	Mean	95% CI
ST1—b	667	63	669	548–783	467	392–592
ST1—b+p	660	83	652	520–851	670	480–920
ST1—b+p+y	743	83	738	593–914	730	607–977
ST1—b+p+y+g	782	84	778	633–966	822	666–996
ST2	4118	319	4142	3423–4666	4768	3185–9198

Analysis using the hierarchical model provides an abundance estimate of approximately 700 female mosquitoes in Z—10 in September 2012, during the low transmission season, with point estimates ranging from 660 to 782, depending on the pooled data. The 95% credible interval varied from 548 to 966 and was the narrowest when the unpooled data (ST1-b) was used, and the widest of all when all cohorts were analyzed together. This might be due to the lowest overall capture ratio when pooling all four cohorts (about 9%) and also due to variation between cohorts. In March 2013 (ST2), during the high transmission season, the estimated abundance of *Aedes aegypti* was four times higher than in the low transmission period, with approximately 4100 female mosquitoes in Z—10. The credible interval (3423 – 4666) is wider than in the low abundance period, but the coefficient of variation is smaller (0.30 for ST2; 0.35 for ST1-b and 0.42 for ST1-b+p+y+g.).

The Fisher—Ford model tended to agree with the hierarchical model (Table 2 and Fig 1). The exception is for the ST1-b data, where the Fisher—Ford point estimate of mosquito abundance is considerably underestimated compared to the other method. Overall, the hierarchical model produced more precise estimates than the ones produced using Fisher—Ford method. This is evident observing the high abundance data (ST2) where the Fisher—Ford confidence interval is 5 times greater than the hierarchical model.

**Fig 2:**
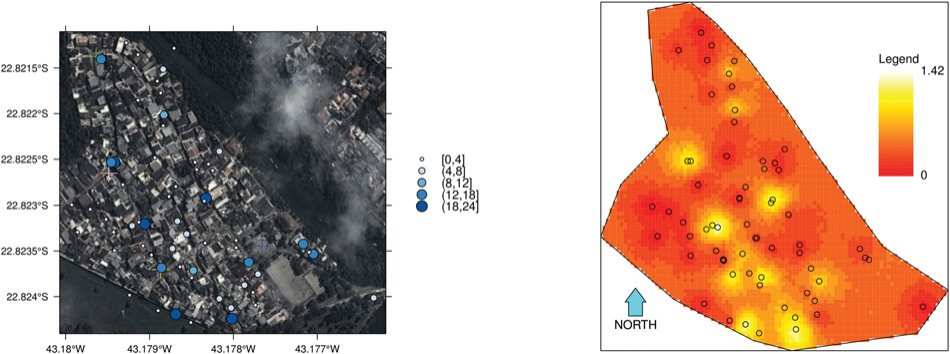
Spatial distribution of individuals in the Z—10 area in Rio de Janeiro, Brazil, in September 2012. Results for ST1 (Sept 2012) obtained with 16,000 iterations (8,000 iterations in each of two chains after a 2,000 burn-in period). Results from analysis using all cohort of marked individuals. Circles indicate trap locations. Bubbles as shown in the maps on the left—hand side indicate the counts of mosquitoes trapped in the MRR experiments. The release points of marked mosquitoes are depicted by crosses, each of which appear in the color of its respective cohort. The spatial density unit is number of mosquitoes per 100 *m* ^2^.

**Fig 3:**
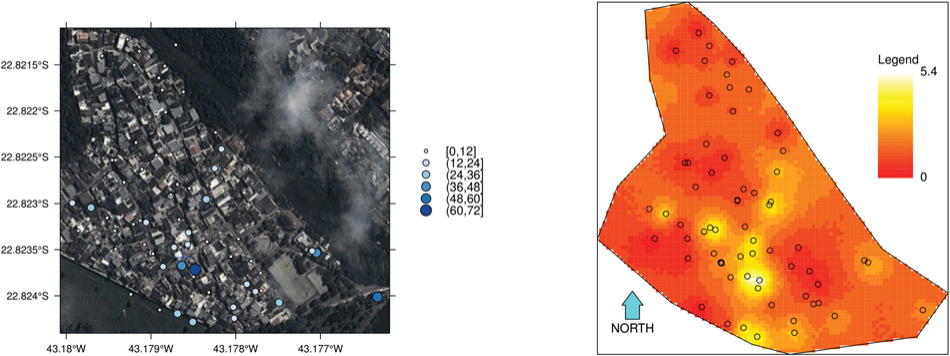
Spatial distribution of individuals in the Z—10 area in Rio de Janeiro, Brazil, in March 2013. Results obtained with 20,000 iterations (10,000 iterations in each of two chains after a 10,000 burn-in period). Results from analysis using the cohort of individuals marked in blue. Circles indicate trap locations. Bubbles as shown in the maps on the left—hand side indicate the counts of mosquitoes trapped in the MRR experiments. A cross indicates the point of releases of marked mosquitoes. The spatial density unit is number of mosquitoes per 100 *m* ^2^.

Analysis using the hierarchical model provides an estimate of the spatial distribution of mosquitoes that is not possible using the Fisher—Ford method. Figs 2 and 3 show the estimated spatial distribution of *Aedes aegypti* in Z—10 in Sept. 2012 (ST1) and March 2013 (ST2), respectively. To facilitate interpretation Figs 2 and 3 have on the left—hand side images of the study area superimposed with a bubble representation of the capture counts per trap and on the right—hand side the posterior spatial density according to the hierarchical model. First, the difference between scales should be noted. In September, local mosquito density peaks at 1.42 mosquitoes per 100 *m* ^2^ while in March, the ceiling is at 5.4 mosquitoes per 100 *m* ^2^. During the high abundance period, mosquito abundance is concentrated in the center—south region.

The survival probabilities for the marked individuals, as estimated by analysis from the hierarchical model, are shown in Table 3. Estimates (mean values) of survival probabilities are expectedly higher for cohort combinations whose capture ratios are also higher.

**Table 3:** Estimates of survival probability for marked population of *Ae. aegypti* released in Z—10, Rio de Janeiro, according to the hierarchical model. Estimation realized using samples from 16000 iterations using data from both studies. The hierarchical model results are obtained using JAGS after running 10000 iterations per chain (first 2000 were discarded) in two Markov chain simulations. Fisher—Ford estimates are obtained using a bootstrap approach (re-sampling *R*_*s*_ = 1000).

	Hierarchical Model			
Cohort	Mean	Std. dev.	Median	95% CI
ST1—b	0.82	0.03	0.82	0.75–0.89
ST1—b + p	0.77	0.03	0.76	0.70–0.83
ST1—b + p + y	0.76	0.03	0.76	0.71–0.82
ST1—b + p + y + g	0.75	0.03	0.76	0.71–0.80
ST2	0.80	0.02	0.80	0.75–0.84

The posterior distributions of abundance and survival probability after analysis of data from both experiments are found in S1 Text.

### Analysis of simulation data

**Fig 4:**
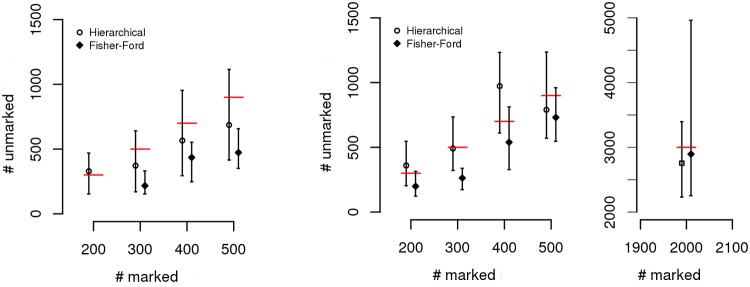
Estimates obtained from MRR data in the simulated scenarios. The estimated values for abundance are shown along the *y*–axis (for all cases in the *y*—axis on the left—hand side, except for the case *N*_2_ = 3000 shown along the *y*—axis on the right—hand side), whereas each case is shown along the *x*-axis, described by the number (known value of) of unmarked individuals used in the simulation. The plot on the left—hand side shows results for a small basin of attraction, whereas on the right—hand side results are shown for traps’ basins of attraction that have radiuses 60% greater. Red lines indicate the numbers of unmarked mosquitoes that were used in the simulations for each of the configurations.

To further compare and understand the behavior of the hierarchical and Fisher—Ford estimates, artificial data from simulated scenarios were analyzed. Table 4 and Fig 4 shows the results obtained from simulations done by varying the population size of marked and unmarked individuals *i.e*., (*N*_1_, *N*_2_): (200, 300), (300, 500), (400, 700), (500, 900), (2000, 3000). Besides the population size, we also compared scenarios with two attraction areas, defined as the radial spatial coverage of each trap. The larger the attraction area, the more attractive the trap is and the higher the chance of capturing mosquitoes. This is a parameter that is very difficult to measure in the field as it depends on the microenvironment and on the trap features. All simulations were done using the concept of center of activity (AC mode in simulator). Table 4 shows the number of captures and the mean and other statistics of the estimated abundance for each simulated scenario. Fig 4 shows the results graphically. In all scenarios, the credible intervals produced from the hierarchical model included the true value. The same was not true for the Fisher—Ford method, which tended to underestimate the true abundance. For the case of a larger basin attraction area the actual numbers *N*_2_ of unmarked mosquitoes are within the intervals given by the hierarchical model, even though the capture ratio of marked individuals is small, approximately 10%. The estimated values found for survival probability and posterior distributions of abundance are found in S1 Text.

**Table 4:** Abundance estimation in simulated scenarios, using the hierarchical and the Fisher-Ford models. The total number of iterations was 12000 for each of 2 Markov chains. The first 3000 iterations are discarded as burn-in interval. The area of attraction is of size 5 (b5) and also 8 (b8), probability *p* = 0.5. The number of traps is *J* = 64. For the Fisher—Ford estimates a number of *R*_*s*_ = 1000 re-sampling was used and the daily survival probability was *ϕ* = 0.8, same value used in the simulations. For the case (200, 300) with a small attraction area the sample size (2) is very small and no Fisher—Ford estimates are reported. The notation (m,u) refers to the quantity (number of captures, capture ratios) for marked and unmarked mosquitoes, respectively.

Simulated scenarios			Hierarchical Model				Fisher—Ford	
simulation	# captures (m,u)	capture ratio	Mean	Std. dev.	Median	95% CI	Mean	95% CI
b5-h / (200, 300)	(5, 29)	(0.03, 0.10)	323	85	329	152–470	—	—
b5-h / (300, 500)	(14, 48)	(0.05, 0.10)	386	128	372	169–642	217	152–332
b5-h / (400, 700)	(17, 74)	(0.04, 0.11)	583	176	566	295–953	435	246–552
b5-h / (500, 900)	(21, 85)	(0.04, 0.09)	705	185	686	414–1116	473	350–658
b8-h / (200, 300)	(17, 64)	(0.09, 0.21)	363	88	360	202–546	199	124–316
b8-h / (300, 500)	(36, 104)	(0.12, 0.21)	501	109	491	322–736	263	172–339
b8-h / (400, 700)	(41, 167)	(0.10, 0.24)	963	164	973	612–1234	539	328–811
b8-h / (500, 900)	(46, 205)	(0.09, 0.23)	818	166	790	570–1237	731	546–961
b8-h / (2000, 3000)	(186, 645)	(0.09, 0.21)	2780	286	2755	2229–3399	2895	2253–4961

## Discussion

Recently proposed strategies [43] to control vector-borne diseases could either reduce the number of individual vector mosquitoes (population suppression) or reduce the competence of these vectors in transmitting the pathogen by replacing the susceptible wild population by genetically modified individuals refractory to the infection by the pathogen (population replacement). Evaluation of the potential success of such interventions requires detailed knowledge of the dynamics of the mosquito population, including its abundance in time and space [8]. In this paper, we present a hierarchical probabilistic model for the estimation of female *Aedes aegypti* abundance from MRR studies. The knowledge of mosquito density from estimation can help in control strategies, at least at a planning phase. The model performs well under simulation conditions and provides more precise estimates than the ones given by the Fisher—Ford method. The proposed model expands previous contributions by Royle *et al*. [17] in adding a survival component and a trap component that models the life span of individuals during MRR experiments and the trapping limitations that make difficult multiple capture events for each of the study individuals. We apply the concept of centers of activity present in [17, 25] to the distribution of a mosquito population within a given area. Clusters of human dengue cases have been identified and associated with certain locations in the community, like neighbor’s homes, local meeting or gathering places and attributed primarily to variation in *Ae. aegypti* population density [44, 45]. The underlying mechanism leading to a positive association between human and mosquito infections at the level of individual houses and neighboring residences [46] is yet not entirely explained and one might speculate that this pattern expresses an indirect evidence of the presence of center of activities for the vector.

This model can be extended in order to account for difference in life—history components, for instance, if using cohorts of different origins. This could be the case of releasing cohorts of mosquitoes especially prepared for control interventions, such as *Wolbachia*—infected mosquitoes. Other extension possibilities include recruitment and survival of unmarked mosquitoes and also influence of gonotrophic cycle in the centers of activity. Such extensions would permit to study important biological relations but would also require more elaborated design in the field experiments to gather data, and by consequence, an added cost of complexity in the analysis.

The method was illustrated with data collected from Z—10, a study area located in a dengue endemic area. According to the hierarchical model’s results, Z—10 had approximately 700 female mosquitoes in the late winter-early spring of 2012, corresponding to a measure of 0.99 mosquitoes/premise (95% CI: 0.80 – 1.23 mosquitoes/premise) or 0.33 mosquitoes/person (95% CI: 0.27 – 0.41 mosquitoes/person). This corresponds to the period of low dengue transmission. In March 2013, during the high transmission season, mosquito abundance jumped to 5.2 mosquitoes/premise (95% CI: 4.3– 5.9 mosquitoes/premise) or 1.8 mosquitoes/person (95% CI: 1.5 – 2.0 mosquitoes/person). The estimated daily survival probability was similar in both seasons, despite the difference in temperature, being between 0.71 and 0.80. This provides an estimate of average life span of 7.5 to 9 days (adding the four days in the laboratory) and is comparable to other values presented in the literature [35]. The analysis using the hierarchical model also provides an estimation of the pattern of spatial distribution of the mosquito population. One can see that in the low transmission season (Sept 2012), there are several hotspots distributed throughout the area, but each one holds an average of approximately 1 mosquito per 100 *m* ^2^. In the high transmission season, local mosquito abundance has a fourfold increase in some areas, and the highest abundance is concentrated in the southern part of the neighborhood. Considering that this is the area with more human movement, the presence of hotspots represents a critical situation for virus invasion and dissemination in this community.

The possibility of estimating spatial density of the population of *Aedes aegypti* is an advantage over methods that consider homogeneous distribution of mosquitoes, such as Lincoln, and Fisher—Ford. The Fisher—Ford method takes survival into account, which is quite important in the case of mosquito populations that have high mortality rates. It is, however, inherently sensitive to a good estimate of survival probability, since an error introduced in the survival parameter might underestimate or overestimate the abundance as a consequence. The attempt at using another capture—recapture method, the Jolly–Seber model, in a package implemented by Laake *et al*. [47] that uses Markov chain Monte—Carlo was not successful because of lack of convergence, due to the insufficient data (low capture ratio, single capture per individual). Such results, taken in a pre—intervention phase, integrated with other tools might be useful in control policies for limiting the spread of the vector.

*Aedes aegypti* larvae were raised using standard rearing protocols, which include low intra- or inter- specific competition, low variations in temperature and high amount of resources. Thus, it is expected that the wing size of lab-reared mosquitoes would be higher when compared to wild mosquitoes, as shown in the results. This would clearly influence dispersal as size has been shown to impact dispersal of individuals, i.e., small females disperse further than larger individuals [27]. However, also in [27], size had no significant impact on survival rates.

Methods for estimating *Aedes aegypti* absolute abundance, other than methods using MRR data, have been proposed in the literature. Williams *et al*. [48] and Jeffery *et al*. [49] used data from comprehensive quantitative pupae surveys to estimate absolute population abundances using life-table models. Since pupal mortality is low, one can compute the mosquito standing crop from the number of containers in the area, number of pupae per container, and adult survival. The latter is generally taken from published MRR studies. The underlying assumptions are that pupal production and survival are stable over time, which rarely hold for more than a few days. An even more detailed life-table model is the classical CIMSIM [50] which requires as input, meteorological, demographic and container availability data. A stochastic model with more than one hundred parameters is required to convert input data into estimates of mosquito abundance [43]. Despite the success of this approach in some settings [50], its large scale application might have drawbacks, especially in areas where the availability and quality of containers varies temporally and/or spatially.

Vector density and survival are key parameters that enter the expression of vectorial capacity $V=\frac{ma^{2}p^{v}}{-\log p}$, where *m* is the density of mosquitoes per humans, *a* is the biting rate, *v* is the average incubation period for the pathogen, and *p* is the survival probability [8]. This expression has guided past control efforts, such as the use of insecticides, and remains the primary benchmark against which new strategies are compared. The methods proposed here make more efficient use of data collected in the field than under current approaches familiar to entomologists and surveillance systems. These models make explicit the sources of uncertainty by allowing the estimation of credibility intervals that accompany each estimate. By casting the estimation process under a formal statistical framework, one can benefit from well established processes of model building which consist of the entertainment of several possible sensible models, parameter estimation in these models, hypothesis testing and deviance analysis, examination of residuals, graphical considerations including displays of the observed and fitted values, and model selection by means of information criteria. This procedure does not guarantee the identification of the “true” model but provides clear steps in model validation through the quantification of uncertainty and the identification of deviations from the premises that entered the formulation of the model. Such formal framework will certainly impact positively on the evaluation process of modern complex intervention strategies that intend to suppress or replace vector populations.

## Supporting Information

S1 Text Hierarchical Model. Description of the components of the hierarchical model, JAGS code, and results including posterior distributions.
(PDF)

S2 Text Simulation Tool. Description of the Individual Based Model and the computational tool that generates data simulating Mark—Release—Recapture experiments.
(PDF)

S1 Dataset Data from Experiment ST1. CSV file that contains data obtained from MRR experiment in September 2012 in the Z10 area in the city of Rio de Janeiro: cohorts and coordinates of capture points (traps) and day of collection.
(CSV)

S2 Dataset Data from Experiment ST2. CSV file that contains data obtained from MRR experiment in March 2013 in the Z10 area in the city of Rio de Janeiro: cohorts and coordinates of capture points (traps) and day of collection.
(CSV)
