# Supplementary material for: A Bayesian Hierarchical Model for Estimation of Abundance and Spatial Density of Aedes aegypti
Source: PLoS One. 2015 Apr 23;10(4):e0123794. doi: 10.1371/journal.pone.0123794 (PMC4408040; doi:10.1371/journal.pone.0123794)
Supplement: S1 Text — Description of the components of the hierarchical model, JAGS code, and results including posterior distributions. (PDF) [file pone.0123794.s001.pdf]

A Bayesian Hierarchical Model for Estimation of Abundance and Spatial Density of *Aedes aegypti*

Daniel A. M. Villela*[1], Claudia T. Codeço[1], Felipe Figueiredo[1], Gabriela A. Garcia[2], Rafael Maciel-de-Freitas[2], and Claudio J. Struchiner[1]

[1]Fundação Oswaldo Cruz, Programa de Computação Científica – Rio de Janeiro, Brazil

[2]Fundação Oswaldo Cruz, Departamento de Entomologia, Laboratório de Transmissores de Hematozoários – Rio de Janeiro, Brazil

Supporting Text File S1 – Hierarchical Model

We describe the study area as $\mathbf{A}$, a convex polygon that comprises any pair of coordinates in which individuals can be found. The total time T is taken in discrete points since observations are regularly spaced in intervals of time, *i.e.*, mosquito traps are observed on a daily basis. There are two kinds of individuals captured by the traps, a *marked* mosquito population released in the area and an *unmarked* native mosquito population. The marked population, of known size N_1, is defined by cohorts given by colors used in the marking process. Let $\mathcal{C}$ be the set of colors used in the experiment and let $\mathcal{M}_c$ be the set of individuals marked with color $c \in \mathcal{C}$. We have the total set of marked individuals $\mathcal{M} = \bigcup_{c \in \mathcal{C}} \mathcal{M}_c$ and the total number of marked mosquitoes be N_1. The estimation model should consider the known number N_1 of marked mosquitoes, and the number of unmarked individuals captured in each of the traps daily in order to do an estimation of abundance, native (unmarked) mosquitoes (N_2) For captured individuals we are able to register their capture histories, *i.e.*, the day it was released and the day it is observed in a given trap. This constitutes the dataset available for estimation. In this dataset, we shall index first the marked individuals, *i.e.*, any i, such that $1 \leq i \leq N_1$, is a marked individual, without loss of generality.

*Correspondence author. Email: dvillela@fiocruz.br

To estimate N_2, that is, the abundance of native mosquitoes in $\mathbf{A}$, a hierarchical model is developed with components: an ecological component describing the dispersal and survival of marked individuals once released, and a observation component describing the capturing of marked and unmarked mosquitoes at traps belonging to a set of traps distributed in the area. The hierarchical structure emerges from the dependence of the observation process on the unobserved ecological process.

Ecological process I: mosquito dispersal

Definition: Center of activity is a subarea of the study area $\mathbf{A}$ inside which the individual will tend to stay. For simplicity we consider it a neighborhood of a point $\mathbf{s_i}$ whose coordinates (s_{xi}, s_{yi}) must be inferred through the analysis. We have the center of activity $\mathbf{s_i} = (s_{xi}, s_{yi})$, for an individual i, $1 \leq i \leq N_1 + N_2$, described by a bivariate position whose prior distribution is a uniform distribution in the area $\mathbf{A}$:

$$\mathbf{s_i} \sim Unif(\mathbf{A}). \tag{1}$$

Ecological process II - survival probability

We describe the probability $\phi_{i,t}$ of a marked individual i to survive from time $t-1$ to t, $1 \leq t \leq T$. Let $q_{i,t}$ describe whether an individual i, $1 \leq i \leq N_1 + N_2$, is present for the study at time t, $1 \leq t \leq T$ (similar to the presence variable described by [1]). We define $q_{i,t} = 1$, if an individual i is present at time t, and $q_{i,t} = 0$, otherwise.

For simplicity, we distinguish how survival is modeled across the two sets of individuals, marked and unmarked ones. For the unmarked population, differently from the marked ones, we certainly have not only death of individuals but also recruitment. Under an assumption that the population is at least temporarily at a stable level in the study area, which remains constant during the period of the MRR study, we consider that the mortality cancels the recruitment component (and possibly an immigration and migration components). As a consequence, the population of (native) unmarked individuals, given the total period T of observation, would remain constant over time, except for the capture of individuals. From a modeling perspective, it means that only the marked population will be affected by the survival component.

The survival component for $q_{i,t}$, $1 \leq i \leq N_1 + N_2$, $1 \leq t \leq T$, builds hierarchically upon its previous presence to be a Bernoulli distribution given the product between the previous presence and its survival in the t–interval, *i.e.* , $q_{i,t}|q_{i,t-1} \sim Bern(\phi_{i,t} q_{i,t-1})$. We should do, however, an adjustment to address observations (by captures) and subsequent removals, when we describe the observation component.

Observation process: trap component

There are J traps placed at known locations identified by $\mathbf{u_j}$, $1 \leq j \leq J$, given each by a pair of coordinates (u_{xj}, u_{yj}). The probability $\pi_{i,j}$ of individual i, $1 \leq i \leq N_1 + N_2$ being captured at trap j is given as a function of distance $d_{i,j}$, the distance from the center of activity $\mathbf{s_i}$ of individual i, $1 \leq i \leq N_1 + N_2$, to the location $\mathbf{u_j}$ of trap j, $1 \leq j \leq J$.

Following [2], this can be treated as a *Generalized Linear Model* (GLM). We choose to use a function that takes a complementary log–log link function of the distance $d_{i,j}$, $1 \leq i \leq N_1 + N_2$ and $1 \leq j \leq J$. Hence, $cloglog(\pi_{i,j})|\mathbf{s_i} \sim \beta_0 + \beta_1 d_{i,j}$, where $d_{i,j}$ is the Euclidean distance from the center of activity of individual i to the location of trap j.

As an individual is captured once at most at any observation time t, we have that the probability of being captured at any trap is given by the product between the probabilities of being "capturable" at trap j, conditioned on its distances to all traps, and its probability that it is captured at j.

Also, a variable $z_{i,t}$ describes a "capturable" trap for an individual i, $1 \leq i \leq N_1 + N_2$, to be caught at time t, $1 \leq t \leq T$. The variable $z_{i,t}$ is described by a categorical distribution that takes the normalized values of $\pi_{i,j}$ from $j = 1$ to $j = J$:

$$z_{i,t}|\pi_{i,j} \sim Cat\left(\frac{1}{\sum_{j=1}^{J} \pi_{i,j}} \pi_{\mathbf{i}}\right). \tag{2}$$

Observation process: capturing component

Let $y_{i,j,t}$ be a variable that describes whether individual i, $1 \leq i \leq N_1 + N_2$ is captured at trap j, $1 \leq j \leq J$, at time t, $1 \leq t \leq T$. The abundance that we want to estimate is defined by the number of unmarked individuals present in the area when the experiment starts. The number of individuals that belong to the unmarked set $\mathcal{U}$ of individuals is given by N_2 at the onset of the study but individuals are removed by the capture process. Therefore for individuals that are

captured, they cannot be observed any longer, since they are removed from the study. Although the unmarked population is considered to be at a constant level, the capture process lowers this level. Once we introduce the removal of mosquitoes by capturing, the survival component becomes:

$$q_{i,t}|q_{i,t-1}, y_{i,1,t-1}, ..., y_{i,J,t-1} \sim Bern(\phi \times q_{i,t-1} \times \prod_{j=1}^{J}(1 - y_{i,t-1})). \tag{3}$$

The observation $y_{i,j,t}$, $1 \leq i \leq N_1 + N_2$, $1 \leq j \leq J$, $1 \leq t \leq T$, is given by a Bernoulli distribution that takes into account the presence in the study of individual i, whether it is capturable at trap j and the probability to be effectively captured at trap j:

$$y_{i,j,t}|\mathbf{s_i}, q_{i,t}, z_{i,t} \sim Bern(\pi_{i,j} \times I_j(z_{i,t}) \times q_{i,t}), \tag{4}$$

where the function $I_j(.)$ is an indicator function to indicate whether $z_{i,t} = j$.

Data augmentation

We use the data augmentation technique as used in [3] to estimate N_2, just adding another layer, a zero–inflated component. We let $M >> N_1 + N_2$ be an overestimated number of individuals that take part in the study. Since we do know the number N_1 of marked individuals, we are effectively adding non–real individuals to the unmarked population. Since these individuals are not observed, this technique effectively adds a zero-inflation component by adding a number of zero entries to the observation data. Let w_i be a variable that indicates whether individual i is real. For the marked population of size N_1, $w_i = 1$, since all individuals are known to exist. For the unmarked population of size $M - N_1$, w_i is a Bernoulli variable with parameter ψ, for which a prior distribution is defined accordingly. As such,

$$w_i \sim Bern(\psi_i), \text{ where} \tag{5}$$

$$\psi_i = \begin{cases} 1 & \text{for } 1 \leq i \leq N_1 \\ \psi, & \text{otherwise.} \end{cases}$$

Once this layer is added to the hierarchical model, the observation variable $y_{i,j,t}$ becomes

$$y_{i,j,t}|\mathbf{s_i}, q_{i,t}, z_{i,t} \sim Bern(w_i \times \pi_{i,j} \times I_j(z_{i,t}) \times q_{i,t}). \tag{6}$$

Table S1.1 shows a list of the components in the model and also a description of the variables and parameters.

Table S1.1: The components of the hierarchical model and description of variables and parameters used in the model.

Components	Description
centers of activities	$\mathbf{s_i} \sim Unif(\mathbf{A})$.
survival component	$q_{i,t} \mid q_{i,t-1}, y_{i,1,t-1}, ..., y_{i,J,t-1} \sim Bern(\phi \times q_{i,t-1} \times \prod_{j=1}^{J}(1 - y_{i,t-1}))$
trap component	$cloglog(\pi_{i,j}) \mid \mathbf{s_i} \sim \beta_0 + \beta_1 d_{i,j}$
	$z_{i,t} \mid \pi_{i,j} \sim Cat(\frac{1}{\sum_{j=1}^{J} \pi_{i,j}} \pi_{\mathbf{i}})$
zero-inflated component	$w_i \sim Bern(\psi_i)$
observation component	$y_{i,j,t} \mid \mathbf{s_i}, q_{i,t}, z_{i,t} \sim Bern(w_i \times \pi_{i,j} \times I_j(z_{i,t}) \times q_{i,t})$

Variables and Parameters	Description
$\mathbf{A}$	Study area
N_1	Number of *marked* mosquitoes
N_2	Number of *unmarked* mosquitoes
M	Total number of individuals
$\mathbf{s_i}$	center of activity of each individual i (coordinates)
$y_{i,j,t}$	capture of individual i at trap j at time t
$q_{i,t}$	presence of individual i at time t
$z_{i,t}$	capturable trap for individual i at time t
w_i	individual i is part of the study
$\pi_{\mathbf{i}} = (\pi_{i,1}, \pi_{i,2}, ...\pi_{i,J})$	probability of individual i being captured at each of the traps
ϕ	daily survival probability of marked individuals
	$\phi \sim Beta(4, 2)$
β_0, β_1	coefficients used in the probability function of the trap capturing process
	$\beta_0 \sim N(0, 0.2)$, $\beta_1 \sim N(0, 0.2)$ (field data)
	$\beta_0 \sim N(0, 1)$, $\beta_1 \sim N(0, 1)$ (simulation data)
ψ_i	probability that individual i is in the study
	$\psi \sim Beta(2, 2)$

Posterior distribution of density estimates

Figure S1.2 shows the results for a simulation in which 10 marked individuals are captured out of 200 total marked individuals. The number used for unmarked individuals was 300.

Figure S1.3 shows the posterior distributions found for the estimation of abundance N_2 and also the survival probability of marked individuals ϕ. The abundance estimate obtained in the second study is more skewed to the higher values, probably due to the large set of data. Indeed the credible interval is large as seen in the table of results.

Figure S1.4 shows a map of the city of Rio de Janeiro that shows the location of the Z-10 area. We also expand on the areas to show Ilha do Governador and the area within which Z-10 is located.

When computing the density values we estimate mean values using samples taken from MCMC runs. The area is divided into a grid, and for each small area density is computed. In the Results section we show the maps that contain the mean density values. We also have for each small area credible intervals of the density. Therefore we are able to show maps that contain the lower and upper limits of these credible intervals. Therefore, in Figure S1.5 we consider the minimum density values and maximum density values. We observe little difference in the spatial distributions by comparing the low–limit values to the high–limit values.

Figure S1.6 shows a sliced view of the spatial distribution for a fixed latitude equal to -22.82268. It is basically the density distribution when considering just a single dimension, in this case the longitude, since latitude is fixed.

Figure S1.7 shows the spatial density of female *Aedes aegypti* in the Z-10 area in Rio de Janeiro, Brazil, using data from MRR experiments conducted in Sept. 2012 and March 2013 in the same scale. Since abundance in September was much lower we see a flattened distribution in Figure S1.7a.

Posterior distribution of survival probability in simulation scenario

Table S1.2 shows the results for the survival probability of marked individuals. The survival probability ϕ tends to be underestimated when the area of attraction is small. In this case the low capture rate of traps seems to impact the ability to accurately estimate the survival probability,

which undoubtedly impacts also in the estimation of abundance as shown in the results for abundance.

Table S1.2: Data obtained for the analysis of survival probability in simulations. The total number of iterations was 12000 for each of 2 Markov chains. The first 3000 iterations are discarded as burn-in interval. The pair (m,u) stands for (marked, unmarked).

Study–Cohort	(N_1, N_2)	Number of captures – (m , u)	Capt. Ratio – (m , u)	Hier. model's mean est.	Std dev.	Median	Conf. Int. (95%)
b5-h	(200 , 300)	(5, 34)	(0.03 , 0.11)	0.71	0.09	0.71	0.53 – 0.88
b5-h	(300 , 500)	(14, 62)	(0.05 , 0.12)	0.73	0.08	0.73	0.57 – 0.87
b5-h	(400 , 700)	(17 , 91)	(0.04 , 0.13)	0.74	0.06	0.74	0.61 – 0.86
b5-h	(500 , 900)	(21 , 106)	(0.04 , 0.12)	0.75	0.06	0.75	0.63 - 0.85
b8-h	(200 , 300)	(17 , 81)	(0.09 , 0.27)	0.80	0.06	0.81	0.68 – 0.92
b8-h	(300 , 500)	(36 , 140)	(0.12 , 0.28)	0.83	0.05	0.83	0.74 – 0.93
b8-h	(400 , 700)	(41 , 208)	(0.10 , 0.30)	0.88	0.04	0.88	0.79 – 0.95
b8-h	(500 , 900)	(46 , 251)	(0.09 , 0.28)	0.75	0.04	0.75	0.67 – 0.84

We also experiment with random movement for individuals. Table S1.3 shows results obtained after applying the Bayesian hierarchical model and the Fisher–Ford method for simulation data generated with settings of random movement for each of the individuals. Mean and median estimates from the Bayesian analysis are close to the actual numbers used in the simulations. However, in the simulation mosquitoes perform each random walks in the space over a few days described by a few points. Since each step of these random walks has a maximum length that is much shorter than the area, we might still find individual centers of activity for mosquitoes even though not formally defined in the simulation (using this option).

Table S1.3: Results obtained after applying the Bayesian hierarchical model and the Fisher-Ford method with data from simulations in which individual movements were set to random movement for both marked and unmarked individuals.

Simulated scenarios			Hierarchical Model				Fisher–Ford	
simulation	# captures (m,u)	capture ratio	Mean	Std. dev.	Median	95% CI	Mean	95% CI
(200, 300)	(18, 63)	(0.09, 0.21)	336	89	329	184 – 524	145	99 – 297
(300, 500)	(30, 115)	(0.10, 0.23)	546	128	531	337 – 830	242	171 – 283
(400, 700)	(36, 165)	(0.09, 0.24)	650	135	629	440 – 970	454	346 – 632
(500, 900)	(44, 204)	(0.09, 0.23)	995	206	974	647 – 1407	625	482 – 1101

JAGS code for the Hierarchical Model

Here we present the JAGS code used for running the Bayesian analysis. The code contains comments, in lines indicated by a # character.

The observation data is entered into an R workspace and is used as an input to JAGS. We typically use multiple chains. The fact that we use an individual model, and observation over traps and time, requires us to develop a three-dimension array as observation data to be used as input to JAGS. In the case of field data, we might have numbers such as approximately 800 individuals caught at traps and 2000 marked individuals released. These large numbers require an even higher number for M to implement the data augmentation, by adding an excess of zeroes in this observation matrix. There are also parameters to be worked in this Bayesian analysis, for instance the positions of each of the centers of activity, values for β_0, β_1, ϕ, ψ. Needless to say, as the number of individuals increases the computing intensity also gets more intense.

```
model {
# Generate priors for beta0, beta1, psi

rbeta0~dnorm(0,1)
beta0 <-rbeta0
rbeta1~dnorm(0,1)
beta1 <- rbeta1
psi~dbeta(2,2)

# Prior for daily survival probability

rrho1 ~ dbeta(4,2)
rho1 <- rrho1
        rho2 <- 0.999

# Generate uniform prior for spatial distribution of
# center of activities
for(i in 1:M) {
       s[i,1]~dunif(Xl,Xu)
s[i,2]~dunif(Yl,Yu)
# w indicates whether individual belongs to the study (zero-inflated component)
w[i] ~ dbern(step(i-(N1+1))*psi + (1-step(i-(N1+1)))*1)
}

# For each individual compute its probability to be present
# at time t from 1 to T
for (i in 1:M) {
```

```
    phi[i] <- step(i-(N1+1))*rho2 + (1-step(i-(N1+1)))*rho1
    q[i,1] <- phi[i]
    for (t in 2:T) {
     q[i,t]<- phi[i]*q[i,t-1]*(1-step(sum(y[i,,1:(t-1)])-1))
}
}

for(i in 1:M) {
pu[i,] <- p[i,]/sum(p[i,1:J])
y3[i]~dcat(pu[i,1:J])

for (j in 1:J) {
# the distance from the center of activity of this individual to the trap location
    d[i,j]<- pow((s[i,1]-X[j,1])^2 + (s[i,2]-X[j,2])^2 , 0.5)
# the probability to be captured at trap j is a function of
# the distance from its center of activity to the trap location
    p[i,j] <-  1-exp(-exp(log(1) - beta0 - beta1*d[i,j]))
for (t in 1:T) {
# The observation as a Bernoulli variable taking into account
# the existence of the individual, its probability to be captured,
# the capturing at trap j, and its survival
    y[i,j,t] ~ dbern(w[i]* p[i,j] * equals(j,y3[i]) * q[i,t])
}

}
}

# The abundance is given by the sum of w[] but we discount
# the number of marked individuals
N<-sum(w[])-N1


}
```

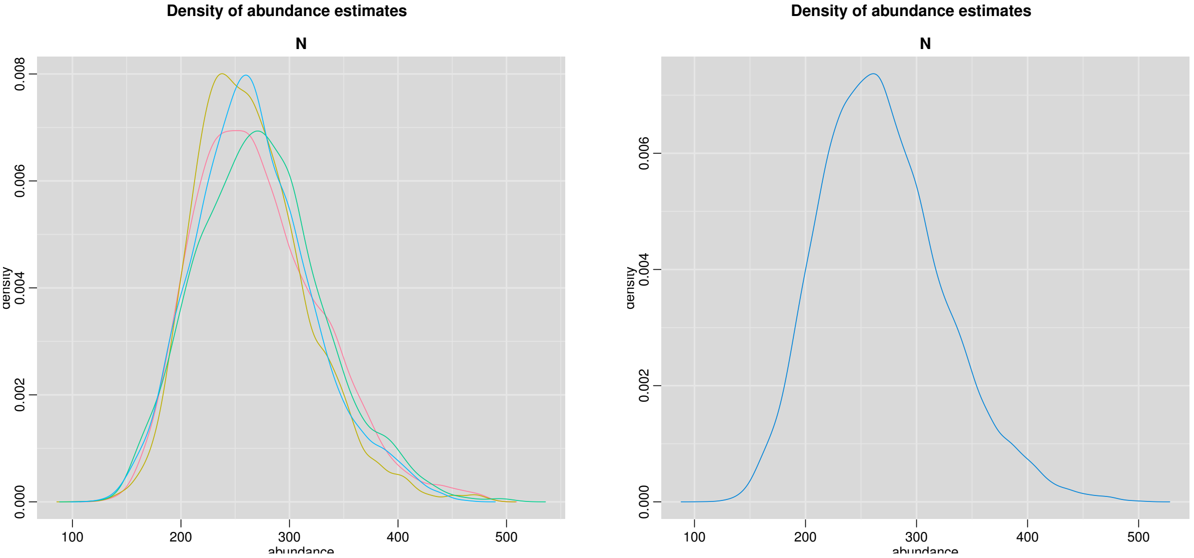

(a) Estimation of abundance (b) Estimation of abundance (collapsed)

Figure S1.1: Results from estimation – using simulation data (number of marked individuals = 200 and number of unmarked individuals = 300). Results obtained after 10000 iterations, 4 chains and a burn-in period of 2000 iterations. Here we run 4 Markov chain simulations and the plots in the boxes on top show the results obtained for each of the simulations. On the left–hand side, results are shown for the estimation with each of the Markov chains. On the right–hand side the posterior distribution for the abundance N_2 is shown, when all distributions for each of the simulations are collapsed into one single distribution for abundance.

References

[1] King R (2012) A review of Bayesian state-space modelling of capture–recapture–recovery data. Interface focus 2: 190–204.

[2] Royle JA, Karanth KU, Gopalaswamy AM, Kumar NS (2009) Bayesian inference in camera trapping studies for a class of spatial capture-recapture models. Ecology 90: 3233–3244.

[3] Royle JA, Dorazio RM (2012) Parameter-expanded data augmentation for bayesian analysis of capture–recapture models. Journal of Ornithology 152: 521–537.

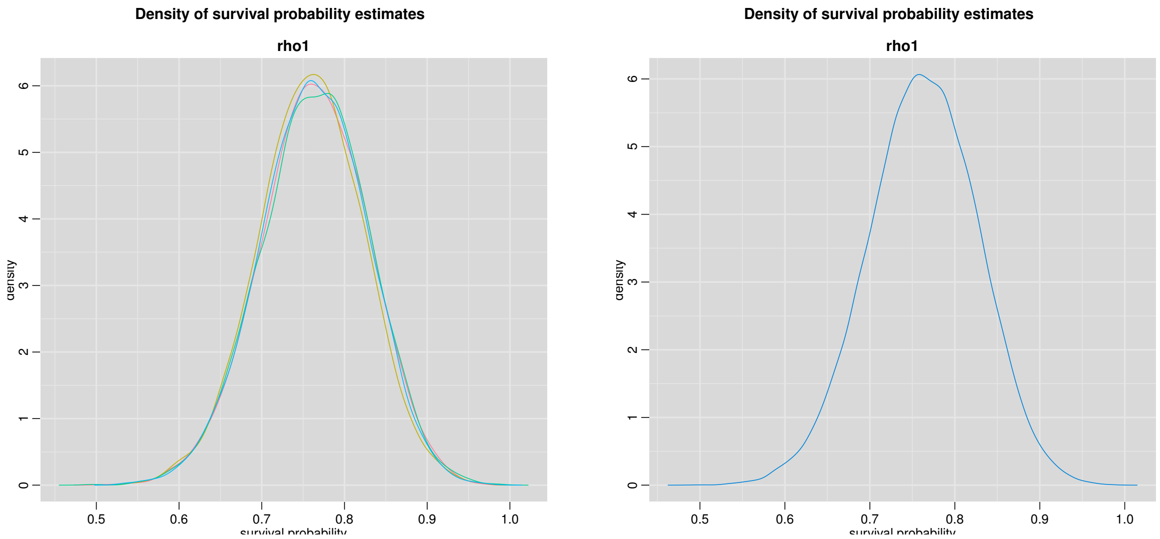

(a) Estimation of survival probability

(b) Estimation of survival probability (collapsed)

Figure S1.2: Results from estimation – using simulation data (number of marked individuals = 200 and number of unmarked individuals = 300). Results obtained after 10000 iterations, 4 chains and a burn-in period of 2000 iterations. Here we run 4 Markov chain simulations and the plots in the boxes on top show the results obtained for each of the simulations. On the left–hand side, the posterior distribution for the survival probability ϕ of marked individuals is shown for each of the Markov chains. On the right–hand side the results are depicted when all distributions for each of the simulations are collapsed into one single distribution of survival probability.

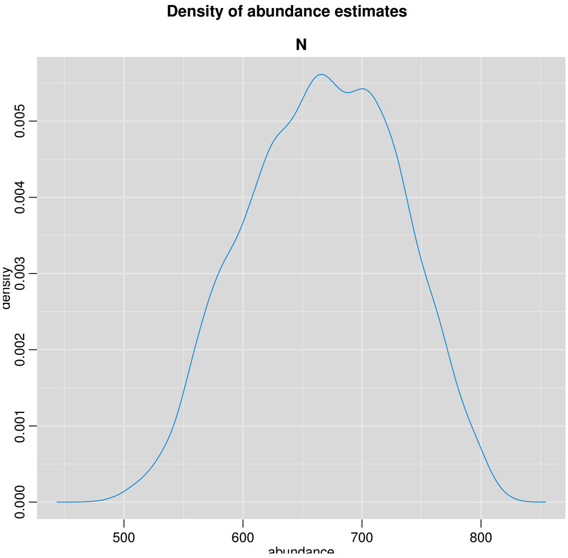

(a) Estimation of abundance (Sept. 2012)

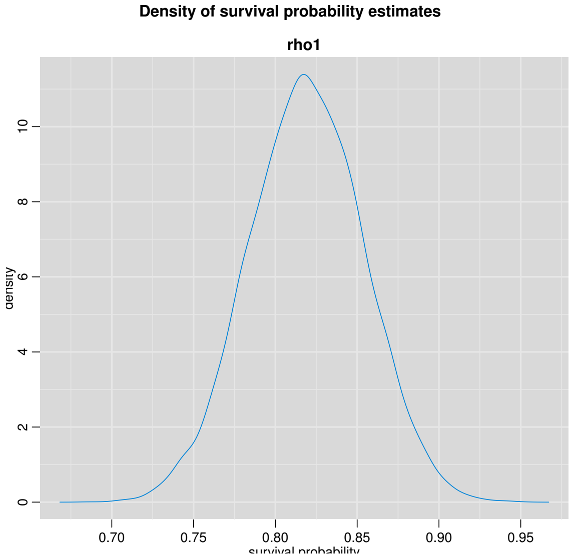

(b) Estimation of survival probability (Sept. 2012)

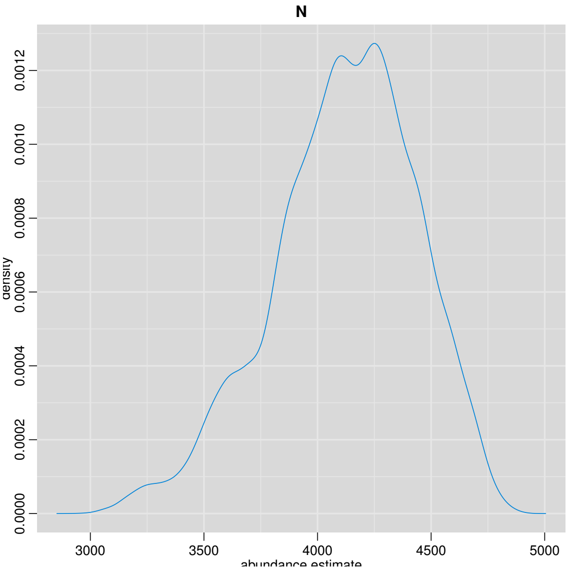

(c) Density of abundance estimates (March 2013)

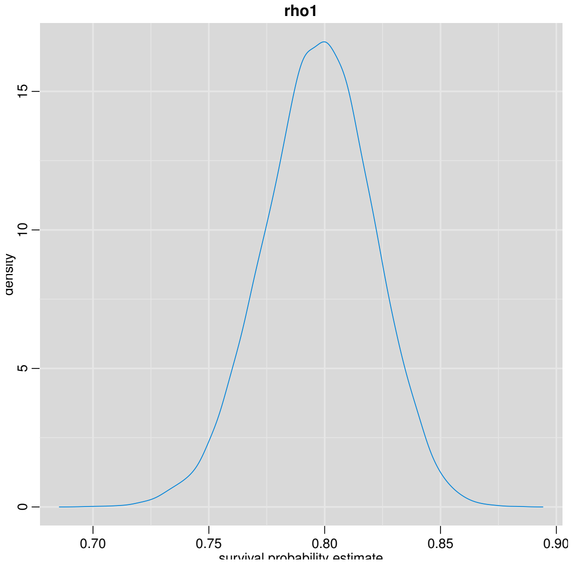

(d) Density of survival probability estimates (March 2013)

Figure S1.3: Posterior distributions of abundance and survival probability after 16000 iterations. On top the distributions for abundance and survival probability found after using the Sept. 2012 data are shown in Figures S1.3a and S1.3b, respectively. In the bottom Figures S1.3c and S1.3d show the distributions for abundance and survival probability, respectively, found when taking into account the data from March 2013.

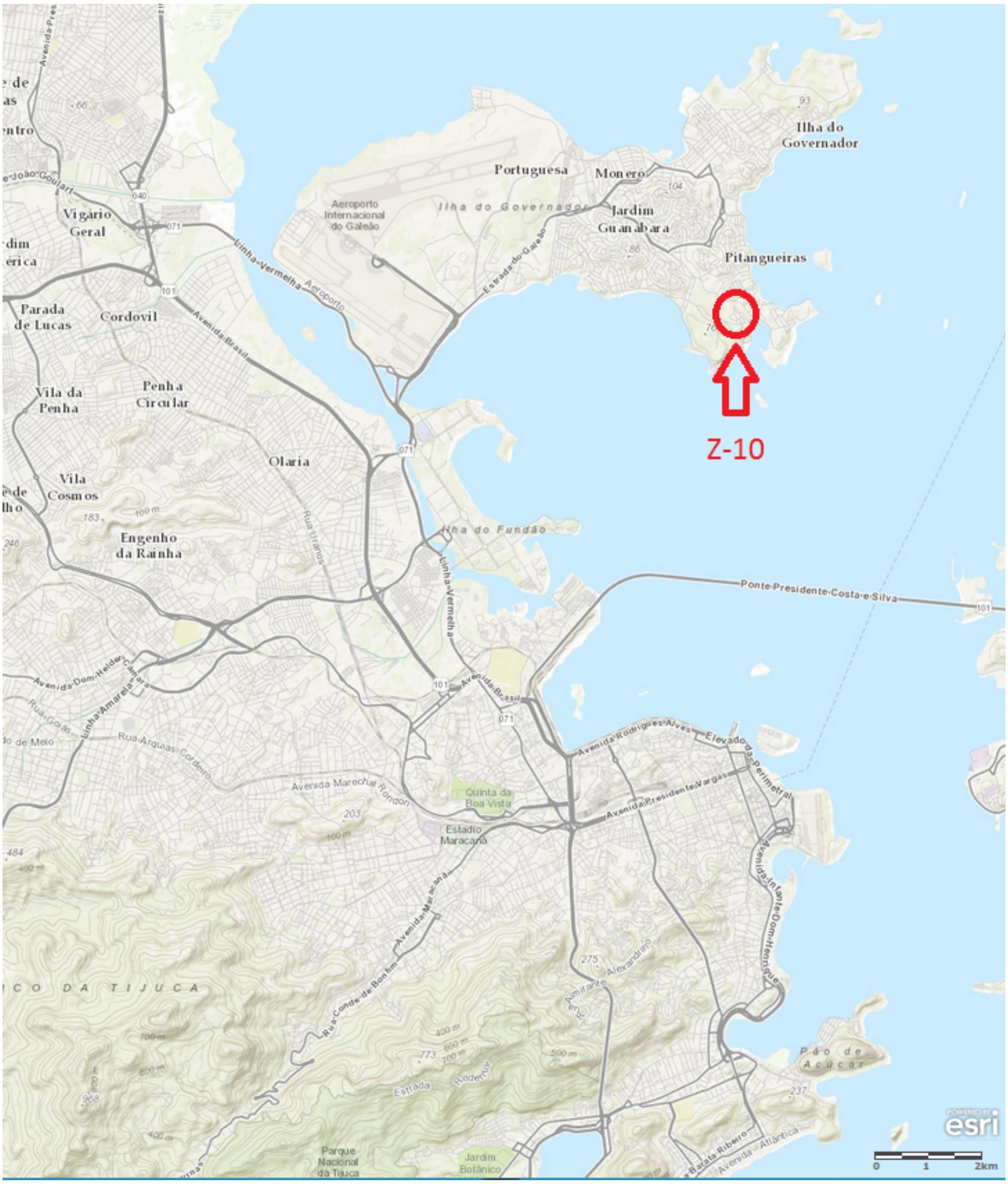

Figure S1.4: This map includes downtown of the city of Rio de Janeiro, a portion of its southern zone, a portion of its northern zone, and the Guanabara Bay on the right–hand side. The Z-10 area is located at Ilha do Governador, which appears in the top right corner. Source of map layer: USGS LandsatLook, http://landsatlook.usgs.gov/

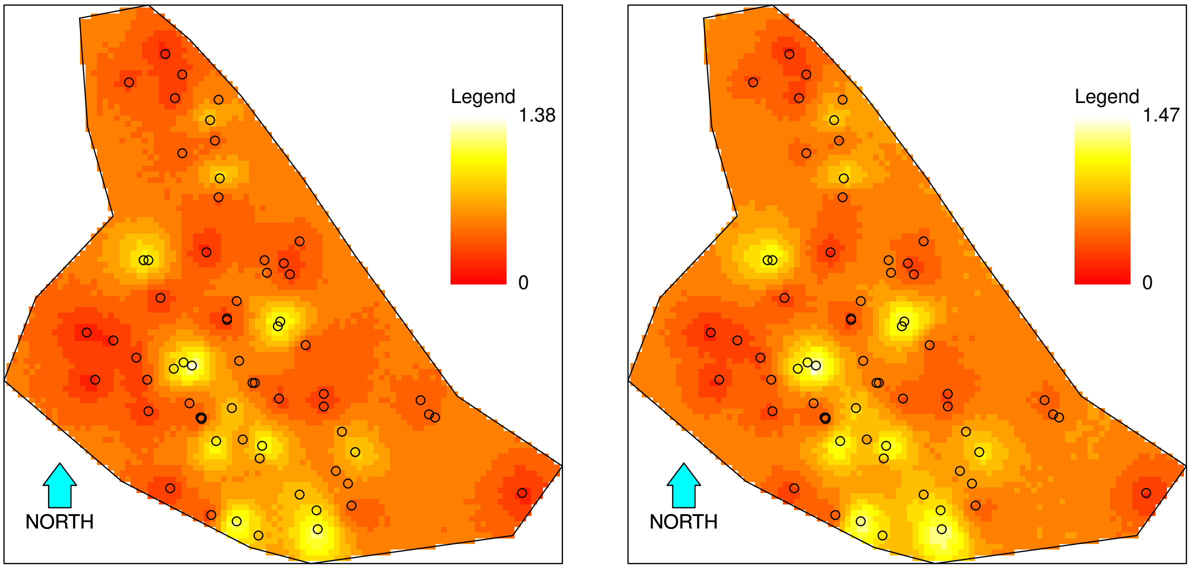

(a) Low–limit values of density estimates

(b) High–limit values of density estimates

Figure S1.5: Spatial distribution of individuals in the area (16000 iterations). Circles indicate trap locations.

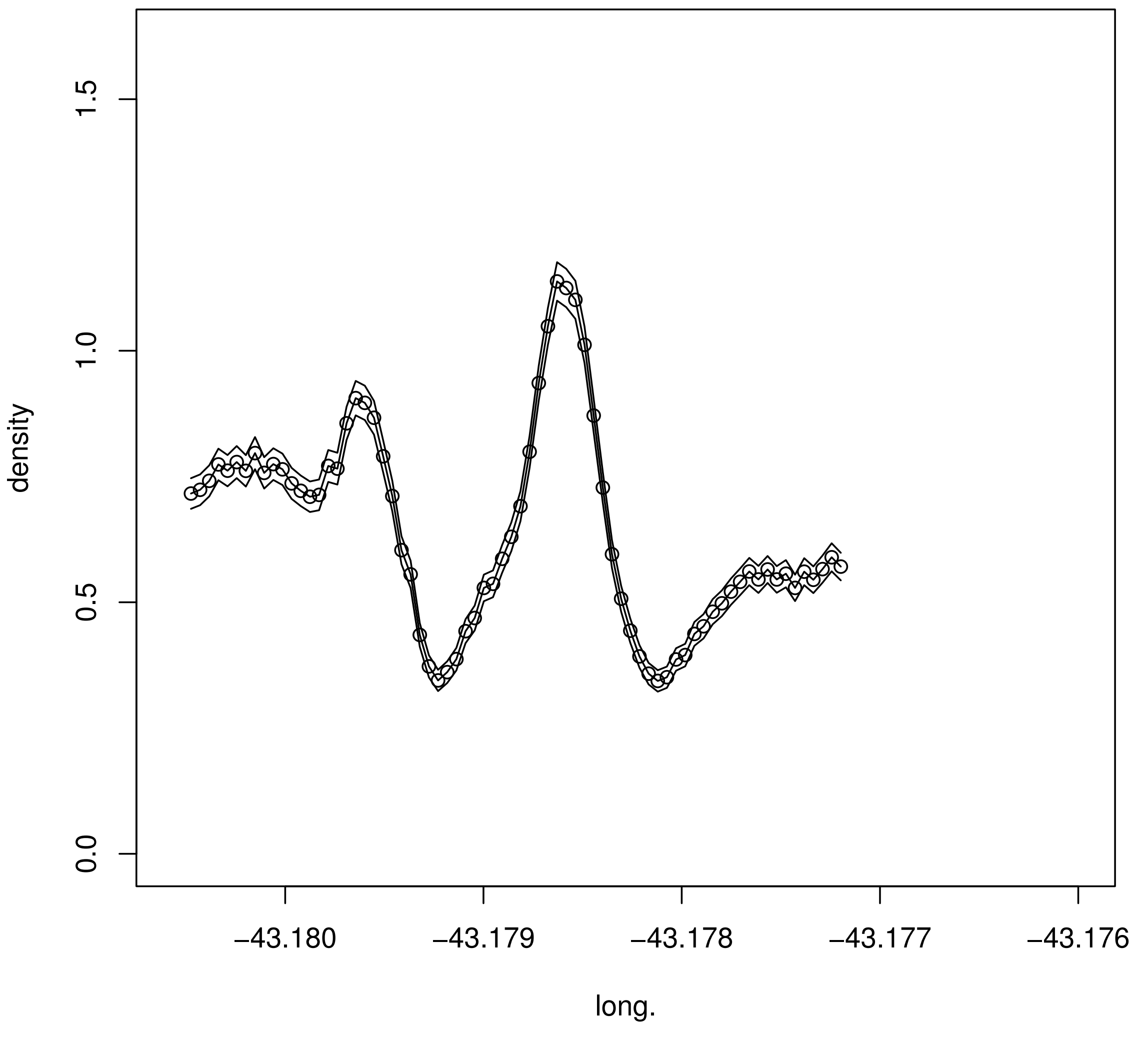

Figure S1.6: Density at latitude = -22.82268. Density is shown in the y−axis for the mean density (number of mosquitoes / 100 m^2). Longitude is shown along the x−axis. The upper and lower limit values from the 95% CI for each gridpiece is shown in the upper and lower curves, respectively.

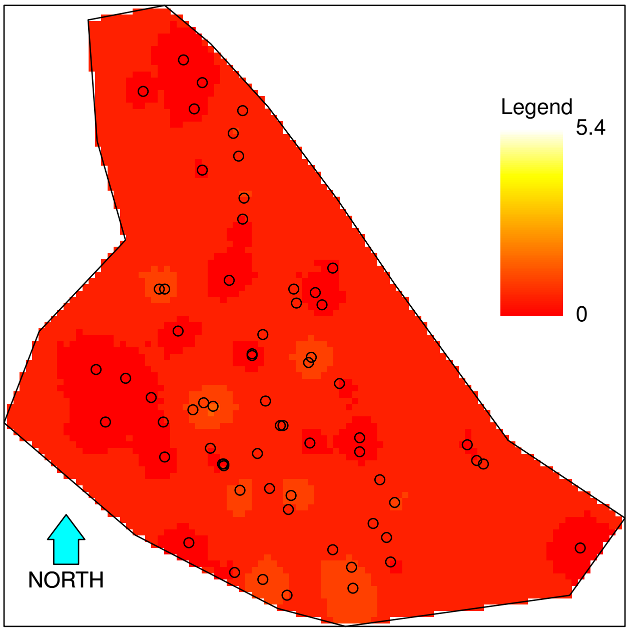

(a) Map with estimation of spatial distribution (Sept. 2012)

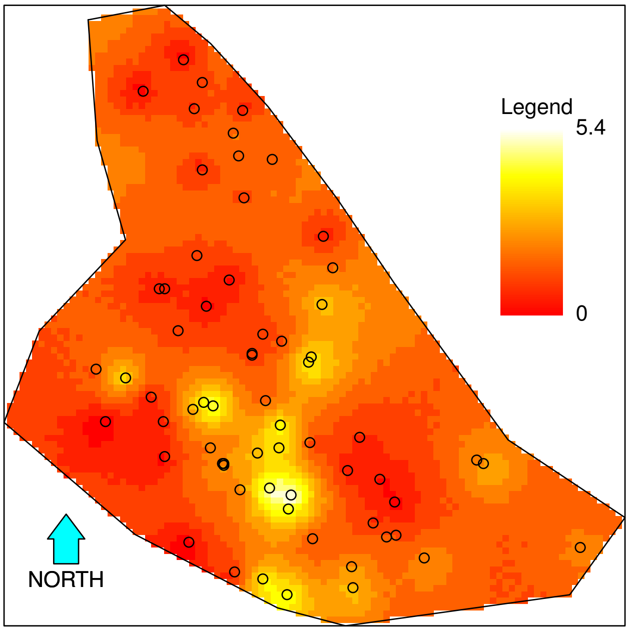

(b) Map with estimation of spatial distribution (March 2013)

Figure S1.7: The spatial density of Sept 2012 compared to March 2013 in the same scale. Circles indicate trap locations.
