# Supplementary material for: A Bayesian Hierarchical Model for Estimation of Abundance and Spatial Density of Aedes aegypti
Source: PLoS One. 2015 Apr 23;10(4):e0123794. doi: 10.1371/journal.pone.0123794 (PMC4408040; doi:10.1371/journal.pone.0123794)
Supplement: S2 Text — Description of the Individual Based Model and the computational tool that generates data simulating Mark—Release—Recapture experiments. (PDF) [file pone.0123794.s002.pdf]

A Bayesian Hierarchical Model for Estimation of Abundance and Spatial Density of *Aedes aegypti*

Daniel A. M. Villela*[1], Claudia T. Codeço[1], Felipe Figueiredo[1], Gabriela A. Garcia[2], Rafael Maciel-de-Freitas[2], and Claudio J. Struchiner[1]

[1]Fundação Oswaldo Cruz, Programa de Computação Científica – Rio de Janeiro, Brazil
[2]Fundação Oswaldo Cruz, Departamento de Entomologia, Laboratório de Transmissores de Hematozoários – Rio de Janeiro, Brazil

Supporting Text File S2 – Simulation Tool

The computational model is designed as an Individual Based Model (IBM), written in the Perl programming language, and divided in independent components or modules, that represent the behavior and interaction among the several agents that compose a Mark-Release–Recapture experiment. A simulation of the experiment is defined by a pre–selected set of parameters, that determine how the rules described below operate on the modeled agents.

S2.1 The simulator

Usage overview

The software execution requires a configuration file `mmrrsim.ini`, in which values for model parameters are defined. The environment is populated with both marked and unmarked mosquitoes, a trap array is set up and then simulation time is counted in discrete units.

*Correspondence author. Email: dvillela@fiocruz.br

General algorithm

At the start of each time step, each available mosquito makes a flight movement inside the spatial grid[1]. If this movement brings it close to a trap, there is a probability that the trap will attract the mosquito and capture it. In this case the mosquito is removed from the population. After all individuals make their daily movement, and all captures are made, the remaining population undergoes a mortality check procedure. The surviving individuals constitute the population in the next time step.

S2.1.1 Description of components

The MMRRSim simulator is composed of four modules: `MMRRSim`, `Mosquito`, `Trap` and `Grid`. The first three are the main classes that define objects that form the dynamics of the simulation, while the last defines the size of the spatial scenario in which the simulation is performed.

All variables were modeled to represent known units of measurement. Each time step represents the interval of time in which all traps are processed by field personnel, all specimens caught are collected and traps are again set up for another capture trial. For MRR experiments carried out with mosquitoes this is typically done on a daily basis. The spatial grid dimensions and the distances travelled by mosquitoes are measured in meters.

Central module

The `MMRRSim` module centralizes the configuration, and the function that calls for all the simulation objects. It provides creation of both `Trap` and `Mosquito` objects, and makes all configured parameters available through the API.

[1] In the following sections we discuss the *spatial grid* that represents the study site in a field experiment, and a *trap grid* contained in it. While we generally make this distinction, for the sake of simplicity we might call the spatial grid simply as *grid*.

S2.1.1.1 Mosquitoes

The `Mosquito` class provides methods for the creation, behaviour and movement rules for each individual in the population. In this section we describe the main attributes of the `Mosquito` objects (Box 1). Both wild (unmarked) and laboratory–reared (marked) subpopulations are represented, and for this model we assume they all have equivalent behaviour, fitness and overall characteristics.

The release of marked mosquitoes can either be simulated to happen in a random location per mosquito or to happen in an unique location for all of them, called *release point*. Unmarked mosquitoes are always created at random locations.

- Name (integer)
- Coordinates (array of two numeric values)
- Survival rate (probability)
- Capture history (binary string)
- Flight autonomy (integer)

Box 1: List of Mosquito attributes, and their respective data types.

The mosquito **name** is an unique (integer) identifier that is used only internally by the program to monitor mosquito location, and as an index in the population set.

Each mosquito's **coordinates** are given by a pair of real numbers (m_x, m_y), and are daily updated by the flight function (described below in section S2.1.1.2).

Each mosquito object can have its own independent attributes[2], and by default all individuals share most of the same attribute values defined by the user in the configuration file, with the obvious exceptions being the identifier (name) and its coordinates.

Survival rate is the probability that the mosquito will not die of natural causes at the end of each day, before being captured by a trap.

The **flight autonomy** parameter determines how far the mosquito can travel daily in each coordinate axis. This does not consider the jitter introduced in each coordinate after the flight

[2]If configured through the simulator API by an *ad hoc* script.

which, in extreme cases, might either extend the distance covered a little further, or cancel out the movement at all (as described in section S2.1.1.2, parameter δ_{max}).

The major advantage of a simulated experiment is its ability to observe information that is not obtainable in the field and can be used to benchmark analysis methodologies. Examples of such informations are the origin and fate of unseen individuals, and actual wild population size. As such, each mosquito created in MMRRSim, either present in the marked or unmarked subpopulation, has its position monitored throughout the simulation time span. The subpopulation of marked mosquitoes starts the experiment with a "captured" indication in its capture history, analogous to a starting cohort in a typical MRR experiment. If it is not recaptured when the experiment ends, it is not computed for analysis. In this case, its capture history only identifies one capture event (the marking event). If at any time it is recaptured by any trap, the mosquito is removed from the population and accounted for in capture data to signal the time of the second capture and final event. This individual data will then be summarized and saved to an output file, as described in section S2.1.1.4.

S2.1.1.2 Flight

Regarding movement of individuals during each time step, we only notice the relevance of two events: possible captures during the time step and, if not captured, its location after its flight movement. This assumption greatly simplifies the system, at the cost of possibly discarding some or most of the complexities of the real phenomenon.

Attraction by a trap does not modify the mosquito actual flight trajectory. Instead, only the position where a mosquito lands at the end of a given day is regarded, and if it stops within a trap's *attraction basin* (section S2.1.1.3), it is assumed that it happened at least once during the time step. In this case the mosquito is a candidate for being captured by that specific trap at that specific day.

There are two flight modes currently implemented in the model: **random flight** and **activity center** (**AC**).

Random flight mode

This flight mode is a typical Markovian movement. At each movement, a random location is

choosen, limited only by the individuals' flight autonomy, and constrained to the spatial grid.

AC mode

While each individual in a natural population might have its own spatial region of preference for its activities, those are heavily influenced by the environment. As such, we take a number of fixed ACs to where all mosquitoes converge. The list of ACs location coordinates can be provided by the user through a CSV file. Upon creation, each `Mosquito` object is attributed one AC from the list uniformly.

Flight error

Regardless of the flight mode choosen for the simulation, a small random quantity (positive or negative) is added to each coordinate to provide further random effects to the dynamics. The maximum magnitude of this quantity is determined by a mosquito attribute provided by the user in the configuration file, called δ_{max}. At the end of each flight calculation, the simulator samples a number $\delta \in (-\delta_{max}, \delta_{max})$, and adds this value δ to the first coordinate. This process is then repeated for the second coordinate.

Constraint to the spatial grid

A movement could take an individual outside of the spatial grid but has to be restrained to the spatial grid. The fact that the grid is a square greatly simplifies the required computing intensity. To check if a given movement remains constrained within the space, it is enough to check if each final coordinate resides in the interval $[0, s]$, where s is the grid side length.

In the case which the movement realizes an unfeasible coordinate, we implemented a *bissection method* by proposing a new coordinate in the middle point that connects the old coordinate and the movement proposal. If that middle point is still unfeasible, we take another half (a quarter of the original distance), and so on, until a feasible coordinate is found. The only assumption made here is that the original location is, indeed, feasible.

S2.1.1.3 Traps

The `Trap` module provides methods for the creation of trap objects and how they interact with mosquito objects. In this section we describe the main attributes and types of `Trap` objects currently

implemented (Box 2). Traps are created at fixed locations and attract mosquitoes that enter a predetermined region around them. Both the amplitude of this region and the efficiency with which they lure a mosquito that is near enough to be captured are defined by these attributes.

In the same fashion of `Mosquito` objects (section S2.1.1.1), most of `Trap` attributes can be set independently on object creation, but in a typical simulation every trap object has equal attributes, except for its name and location.

- Name (integer)
- Coordinates (array of two numeric values)
- Attraction basin (numeric)
- Type (char)
- Efficiency (probability)
- Mosquitoes caught (integer)

Box 2: List of Trap attributes, and their respective data types.

Traps can be created in batch at the start of the simulation, with names and location **coordinates** listed by the user according to the experiment design preferences or constraints. These trap locations can be provided by the user in a CSV file, just as the activity centers. Alternatively, traps can be created in random locations, in which case the only information required from the user is the number of traps to be created.

The trap **name** is a unique identifier that can be monitored in the log file and the CSV tables described in section S2.1.1.4.

Each trap has a disc–shaped neighborhood called **attraction basin** around the trap defined by a radius in meters (Figure S2.1). The radius length is provided by the user by a numeric parameter before the simulation, and is set upon trap creation. A given mosquito is only considered as a candidate for a capture if it is located inside the basin.

The trap **type** selects either a *homogeneous* or a *non–homogeneus* basin. This changes how the capture probability (trap **efficiency**) is calculated within the basin, as described below.

The trap **efficiency** is the probability function p of the distance d between the mosquito and the trap of that trap capturing each mosquito that flies into its attraction basin. A homogeneous trap is defined by a uniform distribution of probability inside the trap basin, in which case $p(d) = c$, for some constant c defined by the user in the configuration file. On the other hand, instead of a constant function of the distance, a non–homogeneous trap uses a complementary log-log function defined by

$$p(d) = 1 - \exp(-\exp(\beta_0 + \beta_1 \times d)), \tag{1}$$

where β_0, β_1 are parameters that determine the probability at the center of the basin ($p(0) = 1 - \exp(-\exp(\beta_0))$) and the decay of the probability as d increases, and both can be selected by the user. Although β_1 determines how fast $p(d)$ decreases, the basin radius described above truncates the attraction basin in order to reduce the number of calculations.

Mosquitoes caught in each trap contribute to the count to be analysed after the experiment is finished. Every individual captured in a given trap is accounted for, and by the end of the simulation, the number of marked and unmarked mosquitoes in each trap is summarized and saved to a file (as described in section S2.1.1.4).

S2.1.1.4 Simulation output

A series of files is produced after the simulation completes successfully, as seen in Box 3

- Log file
- Mosquito data
- Trap data
- Capture histories

Box 3: List of output files.

The **log file** contains the same information printed on screen summarizing the amount of captures that happened at each day. It includes the simulator version, and the objects' pertinent attributes, depending on choices made in the configuration file.

The **mosquito data** file is a table in CSV format containing individual data including AC coordinates and indicators signaling in each entry whether a mosquito was marked (or not), and also whether it was caught by a trap (or not), in which case its coordinates and time of capture are also stored.

The **trap data** file is a table in CSV format that specifies all the traps attributes, including coordinates, how many mosquitoes were caught, and how many of those were marked.

The **capture histories** file is a 'program MARK'–compatible .INP file, summarizing all capture events that occurred during simulation time. All mosquitoes that share the same capture history are grouped into a single line, with the total of the contingent in the second column, with the minus sign implying those individuals were removed from the population upon capturing [1]. After all the capture histories for the recaptured mosquitoes are generated, an additional line is appended, expliciting the remaining mosquitoes that were released and remained unseen during the experiment.

S2.1.2 Default simulation

This section describes the default parameters for simulations. Some parameters were set according to known mosquito ecological values and trap characteristics, while others were inferred empirically to match the data obtained from the Z10 location field experiments. Table S2.1 summarizes the default values for all parameters described below.

In the field experiments, a cohort of 2000 mosquitoes were reared in laboratory conditions from eggs collected at the study site, marked and released in an outdoor point. In a default simulation, much smaller subpopulations are created: 90 unmarked mosquitoes are the wild population in which 10 marked mosquitoes are mixed in order to infer the ecological parameters of interest like abundance, survival and AC locations. We note that, contrary to all other parameters, these default subpopulation sizes are defined for testing purposes. To simplify usage, the user can run a simulation with all default values and select subpopulation sizes via the command line, simply by providing an empty configuration file[3].

[3] The config file must exist at runtime in the current directory and must be called **mmrrsim.ini**.

S2.1.2.1 Environment

The simulation environment is represented by a square, whose side length is determined by the user in the configuration file. The default size is 500m. A central **release point** is used, in coordinates $(250, 250)$.

S2.1.2.2 Traps

In the field experiments, 66 traps were set up, spread over the study site as evenly as possible, inside houses with ininterrupt electricity available for the duration of the experiment. To approximate this, 64 `Trap` objects were set up in an 8 x 8 square, centralized in the spatial grid with equal spaces between them.

In the field experiments, traps are installed inside houses, so the area around each trap are confined to a room or hall, but it is still possible that a mosquito in an adjacent room or just outside a window can detect the trap attractant and fly towards the trap. Thus we postulate that in average the attraction basin of a trap is of 5 meters. This is the value used by default in the simulator.

While it can be hard to determine the actual efficiency of a BG–Sentinel© trap (BioGents, Regensburg, Germany) used in the field, there are reports of both very large amounts of captures of *Anopheles* mosquitoes in open areas in Africa, and very low return rates for marked mosquitoes in Rio de Janeiro (typically 5% total during a week long field experiment, depending on both the density of traps, and population density). For this reason, we also postulate that in average each trap has a default probability of 50% of capturing a mosquito that flies inside its attraction basin. This is the reference value for efficiency in our simulations. For a trap with a homogeneous basin, this probability is constant over distance unless it exceeds the basin radius, in which case the probability of capture drops to zero.

For traps with a non–homogeneous basin, the efficiency at the exact point of the trap (and how it changes over distance) is determined by the parameters β_0, β_1 in function 1. To match the reference efficiency described above, the values for β_0, β_1 were calculated such that over the trap ($d = 0\text{m}$) we have an efficiency of 50% (*i.e.* $p(0) = 0.5$) and at the edge of the basin ($d = 5\text{m}$) the efficiency is lower than 10% (*i.e.* $p(5) < 0.1$). We postulate default values for β_0, β_1 to satisfy

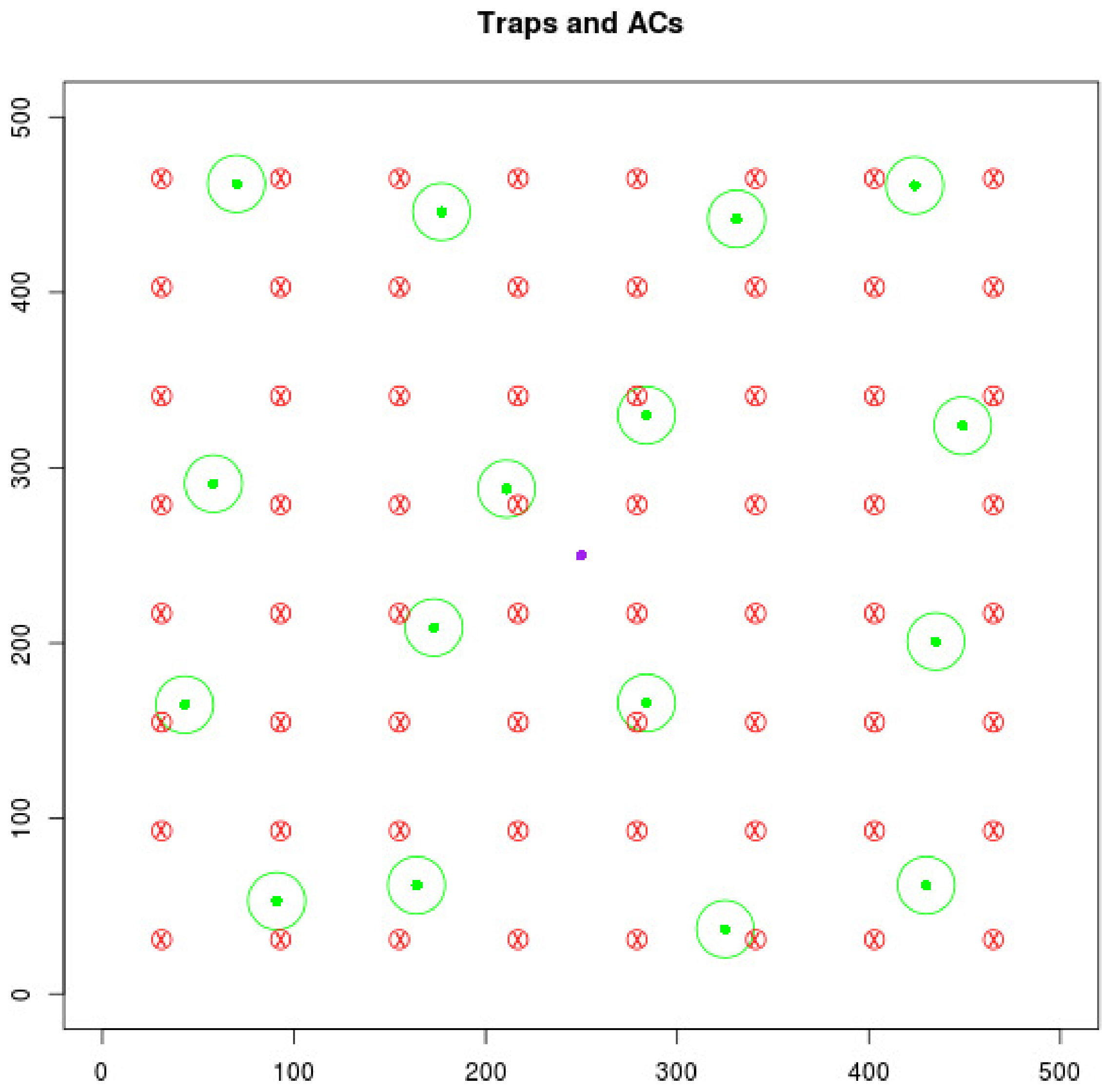

Figure S2.1: Graphical representation of the study site, the traps array and activity centers for a default simulation. Red crosses are trap location, green dots are activity centers, and circles around those indicate the basins of action of these objects (5m radius for traps, and 15m radius for activity centers). The purple dot indicates the release point of marked mosquitoes. Axis, distances and radiuses are represented in meters.

these conditions as closely as possible to the specified bounds.

The **trap type** for a default simulation uses a non–homogeneous basin with $\beta_0 = -0.3665$ and $\beta_1 = -0.5208$, which can be easily shown to satisfy the conditions described above.

S2.1.2.3 Mosquitoes

Each of the attributes described in section S2.1.1.1 are created independently per individual in the simulation, though in a default simulation, all individuals share the same attributes, except location (since each of them fly its own random course after release).

Mosquitoes have a **flight autonomy** of 100m per day, in each spatial coordinate, regardless of flight mode (sections S2.1.1.1 and S2.1.1.2). **Flight mode** is set to AC by default. A list of 16 ACs is provided by default, in a centralized 4x4 array in a similar fashion as the creation of the trap array (section S2.1.2.2). Instead of an uniform array, however, a random jitter was added to each location so as to offer some intersection between AC and traps basins. The default disposition of ACs can be seen in figure S2.1. Together with a daily flight autonomy of 100m, the default random quantity introduced of 15% in mosquito daily flight implies that each AC has a basin of 15m around its center.

The **survival rate** (ϕ) is a fixed rate per mosquito (*i.e.*, constant over time), and to simplify both the model and analysis, in the default simulation all mosquitoes share the same rate. At the end of each simulation step, any mosquito that was not captured by a trap undergoes a Bernoulli draw with $1 - \phi$ as parameter to determine whether it is removed from the population. The default value for the survival rate is $\phi = 0.8$.

The survival rate ϕ always applies to the marked subpopulation, but by default it does **not** apply to the unmarked subpopulation. Whether it also applies to the unmarked subpopulation can be set by the **unmarked reset** parameter. If this option is set to *true*, the unmarked mosquitoes do not undergo a mortality check, and the same amount as the unmarked individuals captured is replenished, to maintain subpopulation size constant.

Table S2.1: Values for parameters in a default simulation. Parameters marked with an asterisk (*) are not configurable through the input file.

parameter	default value
MMRRSim	
release point*	(250,250)
max time	10
unmarked subpop.	90
marked subpop.	10
unmarked reset	no
Grid	
side	500m
Mosquito	
survival rate (ϕ)	80%
flight autonomy	100m
delta_max	15%
flight mode	AC
# of ACs*	16
proportion to AC	100%
Trap	
type	non–homogeneous
attraction basin	5m
homogeneous efficiency	50%
non–homogeneous β_0	-0.3665
non–homogeneous β_1	-0.5208
# of traps*	64
array distribution*	uniform + jitter

S2.2 Validation of the simulator

In order to verify that the assumptions made and the algorithms developed reliably approximate the field experiments, we postulated some of the default parameters for `Trap` objects to approximate a hypothethical trap with a 5m radius, as described in section S2.1.2.2. To this end, we adjusted

both the trap efficiency and marked and unmarked population sizes.

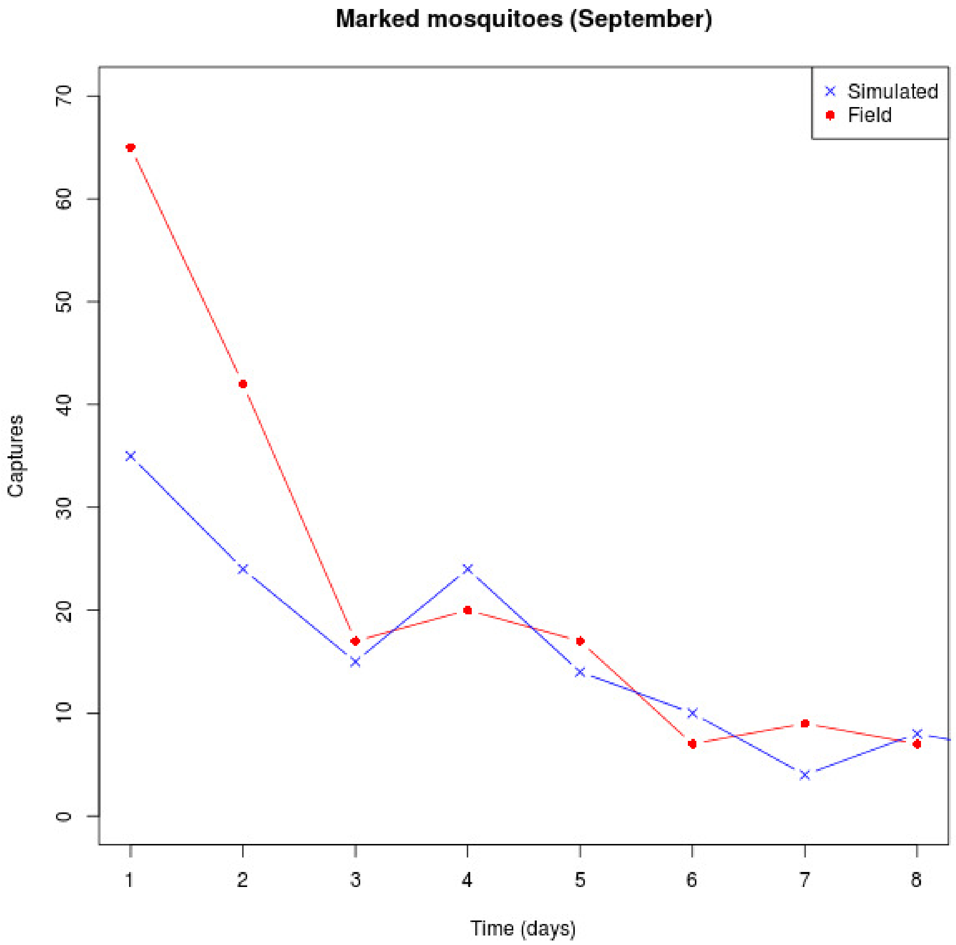

Figure S2.2: Daily captures for marked and unmarked mosquitoes in traps both in the field (red dots) and simulated (blue crosses) experiments. These are results for the marked mosquitoes in the September data set.

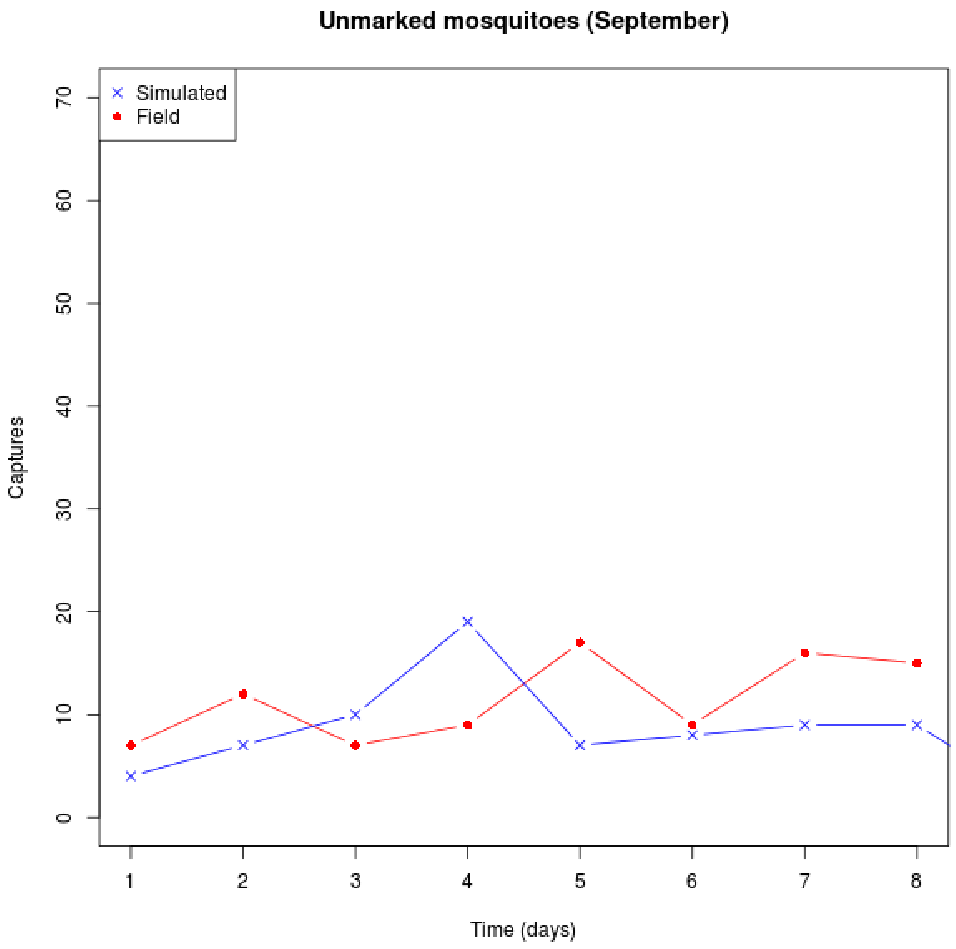

Figure S2.3: Daily captures for marked and unmarked mosquitoes in traps both in the field (red dots) and simulated (blue crosses) experiments. These are results for the unmarked mosquitoes in the September data set.

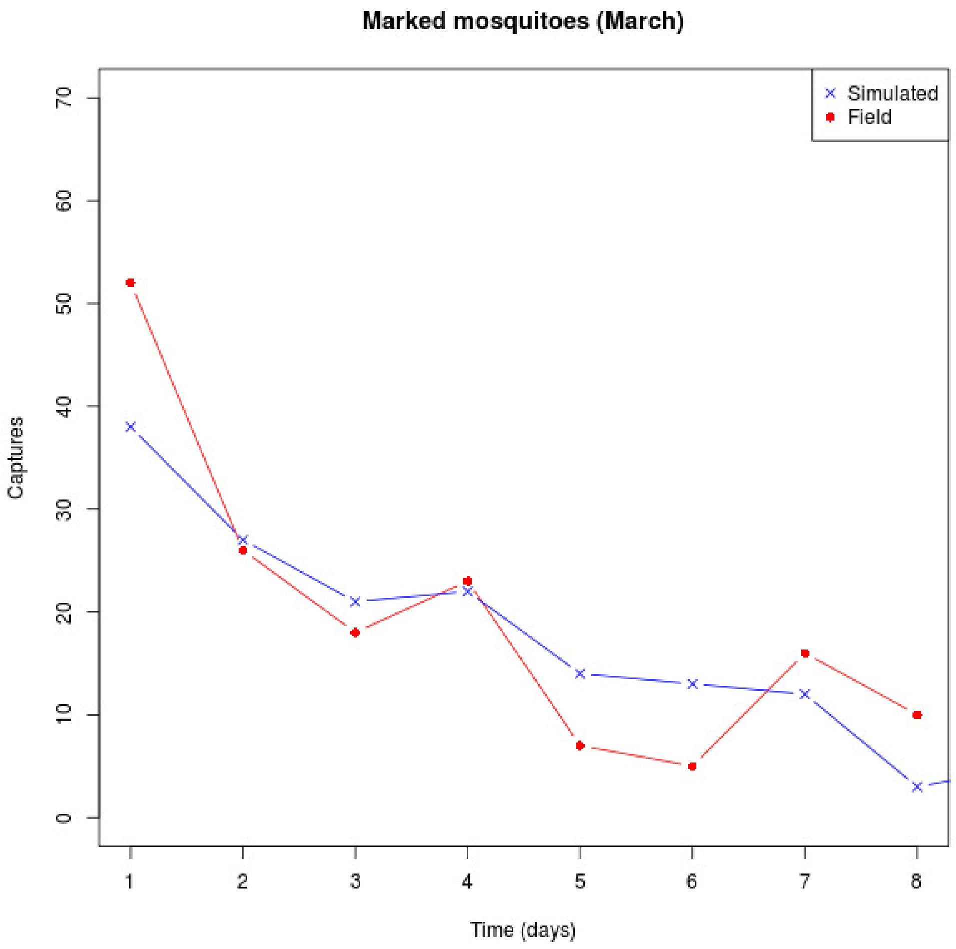

Figure S2.4: Daily captures for marked and unmarked mosquitoes in traps both in the field (red dots) and simulated (blue crosses) experiments. These are results for the marked mosquitoes in the March data set.

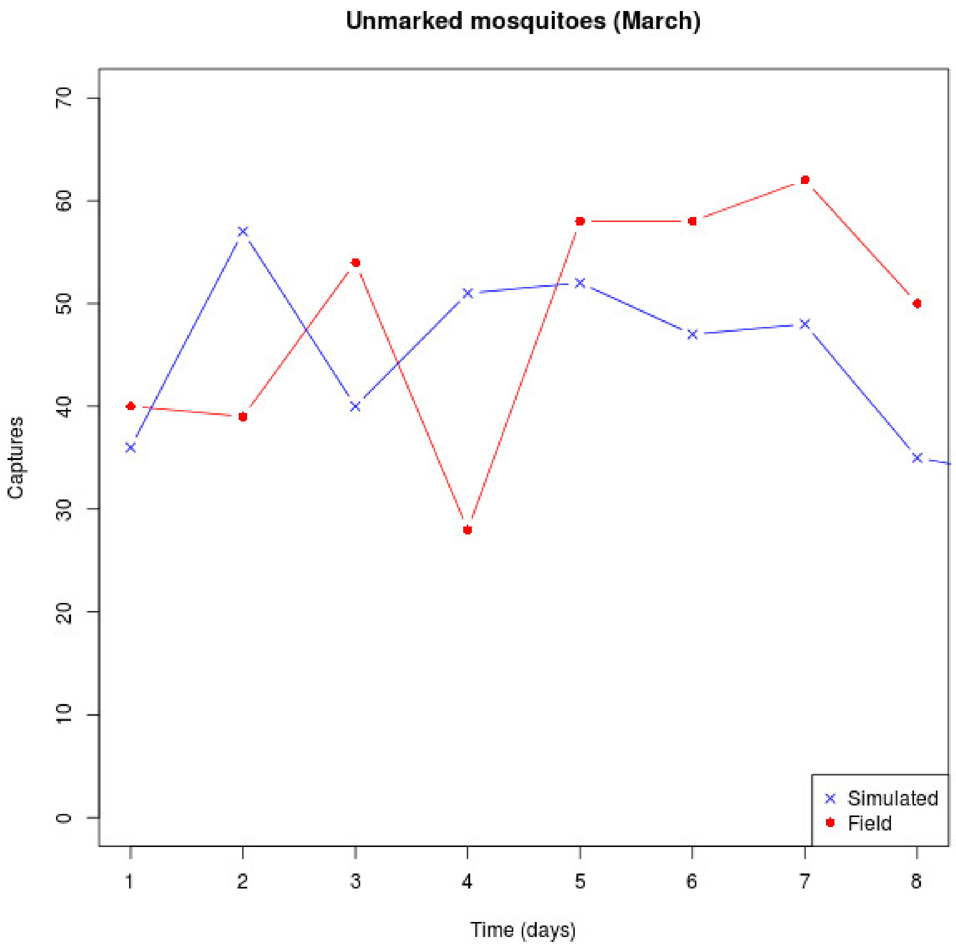

Figure S2.5: Daily captures for marked and unmarked mosquitoes in traps both in the field (red dots) and simulated (blue crosses) experiments. These are results for the unmarked mosquitoes in the March data set.

Figures S2.2 and S2.3 show time series of simulated data and field data from the field experiments performed in September. For this simulation, we used trap efficiency 80%, 3000 marked mosquitoes with a wild population of 600 mosquitoes. Figures S2.4 and S2.5 show the analogous simulation for the March field data, where we simulated trap efficiency of 80%, 4000 marked mosquitoes and a wild population of 3500 wild mosquitoes.

S2.3 Conclusion

Individual-based models permit to construct a software tool by setting rules that are only constrained to computational intensity and computational resources, such as memory usage. Applying even simple rules to individuals, as compared to groups of individuals such as in models based on differential equations, can make complex, realistic behaviors to emerge.

The results in section S2.2 show that this model is able to reproduce the field experiment it proposes to, freeing field staff from the cost and labor of testing small alterations to an experimental design before knowing the impact on the results. These simulations were also used in the present work to calibrate the stochastic model in order to obtain better estimates from the data generated from the field.

The source code, bug tracking and support channel are hosted at `https://launchpad.net/mmrrsim`. The software is released in the open-source license GPL (General Public License).

References

[1] Cooch E, White G, editors (2013) Program MARK: A Gentle Introduction. 12 edition. URL `http://www.phidot.org/software/mark/docs/book/`.
